# Loss of Cx43 in Murine Sertoli Cells Leads to Altered Prepubertal Sertoli Cell Maturation and Impairment of the Mitosis-Meiosis Switch

**DOI:** 10.3390/cells9030676

**Published:** 2020-03-10

**Authors:** Erika Hilbold, Ottmar Distl, Martina Hoedemaker, Sandra Wilkening, Rüdiger Behr, Aleksandar Rajkovic, Marion Langeheine, Kristina Rode, Klaus Jung, Julia Metzger, Ralph H. J. Brehm

**Affiliations:** 1 Institute of Anatomy, University of Veterinary Medicine Hannover, Foundation, 30173 Hannover, Lower Saxony, Germany; 2 Institute for Animal Breeding and Genetics, University of Veterinary Medicine Hannover, Foundation, 30559 Hannover, Lower Saxony, Germany; 3 Clinic for Cattle, University of Veterinary Medicine Hannover, Foundation, 30173 Hannover, Lower Saxony, Germany; 4 Platform Degenerative Diseases, German Primate Center, Leibniz Institute for Primate Research, 37077 Göttingen, Lower Saxony, Germany; 5 Department of Pathology, University of California San Francisco, San Francisco, CA 94143-0794, USA; 6 Department of Obstetrics, Gynecology and Reproductive Sciences, University of California San Francisco, San Francisco, CA 94143-0794, USA; 7 Institute of Human Genetics, University of California San Francisco, San Francisco, CA 94143-0794, USA

**Keywords:** Cx43, impaired spermatogenesis, mitosis-meiosis switch, Sertoli cell maturation, NGS

## Abstract

Male factor infertility is a problem in today’s society but many underlying causes are still unknown. The generation of a conditional Sertoli cell (SC)-specific connexin 43 (Cx43) knockout mouse line (SCCx43KO) has provided a translational model. Expression of the gap junction protein Cx43 between adjacent SCs as well as between SCs and germ cells (GCs) is known to be essential for the initiation and maintenance of spermatogenesis in different species and men. Adult SCCx43KO males show altered spermatogenesis and are infertile. Thus, the present study aims to identify molecular mechanisms leading to testicular alterations in prepubertal SCCx43KO mice. Transcriptome analysis of 8-, 10- and 12-day-old mice was performed by next-generation sequencing (NGS). Additionally, candidate genes were examined by qRT-PCR and immunohistochemistry. NGS revealed many significantly differentially expressed genes in the SCCx43KO mice. For example, GC-specific genes were mostly downregulated and found to be involved in meiosis and spermatogonial differentiation (e.g., *Dmrtb1*, *Sohlh1*). In contrast, SC-specific genes implicated in SC maturation and proliferation were mostly upregulated (e.g., *Amh*, *Fshr*). In conclusion, Cx43 in SCs appears to be required for normal progression of the first wave of spermatogenesis, especially for the mitosis-meiosis switch, and also for the regulation of prepubertal SC maturation.

## 1. Introduction

A steady decrease in birth rate is one of the most severe social problems in industrialized countries nowadays [1]. In this context, a significant decline in male reproductive function has been reported in recent years [2,3,4] and approximately 7% of all men are affected by infertility [5]. However, today, in about 50% of male factor infertility cases, the underlying causes are largely unknown [5].

Intercellular junctions, especially gap junctions, might be interesting approaches regarding the development of non-obstructive azoospermia, as recent studies have shown [3,6,7,8,9].

Gap junctions consist of two hemichannels, termed connexons, which are formed by six structural proteins, the connexins (Cxs), and connect the plasma membranes of two neighboring cells, allowing direct cell-to-cell communication by granting the passage of small molecules (<1 kDa) and ions in various tissues [10,11,12,13]. Consequently, they regulate several physiological functions such as cell proliferation, differentiation, apoptosis and, among other things, they are involved in embryogenesis, electrical coupling, metabolic support, enhanced tissue response and homeostasis [12,14,15]. A total of 20 different Cxs in the mouse and 21 in the human have been discovered since 2004 [16].

In the testis, Cx43 is the predominant gap junctional protein and pubertal differentiation marker [6,7,17,18]. It is located between germ cells (GCs) and Sertoli cells (SCs) as well as between adjacent SCs in the seminiferous epithelium [17,18,19,20,21,22,23,24]. Furthermore, its expression is known to be an absolute requirement for normal testicular development and successful spermatogenesis [25,26]. Alterations of its expression are associated with various testicular disorders in men, e.g., spermatogenic arrest at the spermatogonial level, SC-only (SCO) syndrome and the transition from preinvasive GC neoplasia in situ (GCNIS) to seminoma [6,7,8,27,28,29].

By generating a conditional SC-specific Cx43 knockout mouse line (SCCx43KO), an innovative translational mouse model has been created providing a good opportunity to elucidate numerous mechanisms underlying human male infertility [25,26].

The first wave of normal murine spermatogenesis is ‘initiated’ shortly after birth [30,31]. B-spermatogonia are present at day eight post-natum (p.n.), undergo the last mitotic division to form preleptotene spermatocytes which undergo a final replication of nuclear DNA and then enter the meiotic prophase I by Day 10 p.n. [32]. Adult SCCx43KO males show an arrested spermatogenesis at the spermatogonial level or SCO similar to human phenotypes. Additionally, it has been demonstrated that the first wave of spermatogenesis is inhibited, resulting in infertile SCCx43KO males [25,26]. A previous microarray study [9] examining testes of 8-day-old wild type (WT) and knockout (KO) mice detected plenty of significantly differentially expressed genes in the SCCx43KO mice. The majority of altered genes are associated with spermatogenesis, downregulated, GC-specific and involved in meiosis of male GCs. Among these candidate genes are genes belonging to the doublesex and mab-3-related transcription factor (DMRT) gene family, such as *Dmrtb1* (FC -68.15) and *Dmrtc2* (FC -7.31), as well as *Sohlh1* (FC -6.98) and *Ovol1* (FC -2.4) [9].

These new murine candidate genes could represent helpful markers for exploring human testicular biopsies from patients showing corresponding spermatogenic deficiencies and for studying the molecular mechanisms of human male sterility.

In accordance with data obtained from mice [33], a relevant role in coordinating the transition between mitosis and meiosis has been shown for DMRTB1 in men, as well as a correlation between an altered expression pattern of DMRTB1 in patients suffering from spermatogenic arrest at the level of spermatogonia and mitosis as well as the transformation into B-spermatogonia [34].

Thus, the present study aimed to further analyze the underlying molecular mechanisms and possible signaling pathway(s) of 8-, 10-, and 12-day-old WT and KO mice by next-generation sequencing (NGS) using mRNA-seq, qRT-PCR as well as immunohistochemistry (IHC) and to identify promising candidate genes from SCCx43KO mice for further investigations in corresponding deficiencies using human testicular biopsies.

NGS revealed many significantly differentially expressed genes in the prepubertal SCCx43KO mice compared with their WT littermates, confirming and extending the previous study [9]. As expected, most significant differences were found between the 10-day-old age groups concomitant with the first appearance of spermatocytes in the WT mice. In general, in the SCCx43KO animals, GC-specific genes were mostly downregulated and found to be involved in meiosis and spermatogonial differentiation (e.g., *Dmrtb1*, *Sohlh1*), whereas SC-specific genes implicated in SC maturation and proliferation were mostly upregulated (e.g., *Amh*, *Fshr*). In summary, these findings indicate a crucial role for Cx43 in SCs for normal progression of the first wave of spermatogenesis, in particular for the transition between mitosis and meiosis of GCs, as well as for the regulation of prepubertal SC maturation.

## 2. Materials and Methods

### 2.1. Generation of SCCx43KO Mice

The conditional KO mouse line, which lacks Cx43 solely in SCs, was generated using the Cre/loxP recombinase system. The detailed breeding strategy as well as genotyping and confirmation of Cx43 loss by β-galactosidase IHC were described previously [26]. All husbandry and experimental procedures were conducted according to the German Animal Protection Law and approved either by the Animal Rights Committee of the Regional Commission of Giessen (decision V54-19 c 20/15 c GI 18/1) or the institutional ethics committee and the Lower Saxony State Office for Consumer Protection and Food Safety (reference numbers 33.9-42502-04-12/0877 and AZ 33.19-42502-05-16A017).

### 2.2. Tissue Sampling and Treatment

Animals at different postnatal ages (8, 10 and 12 days p.n. and older than 75 days p.n. (hereinafter referred to as adult)) were anesthetized by CO_2_ and then sacrificed by cervical dislocation. Both testes or the ovaries were removed immediately from each mouse. One testis was either fixed in Bouin’s solution (10% formaldehyde, 4% picric acid, 5% acetic acid) for 48h and subsequently transferred to 70% ethanol followed by paraffin embedding according to standard techniques or snap-frozen in liquid nitrogen and then stored at −80 °C until RNA extraction for NGS and qRT-PCR. Ovaries were also fixed in Bouin’s solution, transferred to 70% ethanol and embedded in paraffin. Testes and ovaries of adult mice were solely used for IHC.

### 2.3. Histochemical Techniques and ‘Cell Counting’

For morphological evaluation and IHC, 3-µm slices of Bouin-fixed paraffin-embedded testes from all KO and WT mice were sectioned. Hematoxylin and eosin (H&E) staining was performed according to standard techniques. On the one hand, the mean number of SCs per seminiferous tubule of the KO mice was found to be significantly increased in pubertal and adult KO mice compared with their WT littermates, while, on the other hand, the mean GC number was significantly reduced in 8-day-old KO mice [9,25,26]. To determine GC and SC numbers per tubule cross-section in prepubertal animals, SOX9 IHC (SC marker, see Chapter 2.4) was conducted and then GCs and SCs of 8-, 10- and 12-day-old KO and WT mice (*n* = 4 per age group and genotype) were counted as described previously [26,35] using a ‘Zeiss Axioskop’ microscope (Zeiss, Jena, Germany), ‘Olympus DP 70′ camera (Olympus, Hamburg, Germany) and ‘Olympus DP Soft’ software (V 3.2).

### 2.4. Immunohistochemistry

Immunolabeling was conducted on Bouin-fixed and paraffin-embedded testicular sections, which were mounted on silane-treated glass slides (Histobond Superior; Paul Marienfeld Laboratory Glassware, Laud-Königshofen, Germany) and dried at 37 °C for 24 h.

In order to confirm successful Cx43 gene deletion and Cx43 protein loss, β-galactosidase and Cx43 IHC were performed. SOX9 immunolabeling was conducted to mark SCs and thus to ensure a clear identification of these cells for cell counting (see above). Moreover, immunostainings for AMH, LIN28A, SALL4 and SOHLH1 were carried out to examine the protein expression of the selected genes that were found to be significantly regulated either by NGS or by NGS and qRT-PCR in prepubertal KO mice.

All antibodies used in the present study are summarized in Table 1. For negative controls, the same protocols were used but the primary antibody was either omitted and replaced by a polyclonal anti-rabbit IgG antibody (Sigma Aldrich, München, Germany) and/or substituted by buffer.

After paraffin-embedding and sectioning, the slides were deparaffinized and rehydrated. To block the endogenous peroxidase, all sections were incubated in 196 mL 80% alcohol comprising 4 mL hydrogen peroxide at room temperature for 30 min. Following this, heat-induced antigen retrieval was performed using sodium citrate buffer (pH 6.0) and a heat plate at 96 to 99 °C for 20 min (SOX9, AMH) or slides were either microwaved at 800 watts for three minutes and 12 min at 600 watts (SOHLH1) or at 800 watts for 15 min (Cx43) or for 10 min (LIN28A, SALL4). β-galactosidase IHC was conducted without antigen retrieval. Next, the slides were incubated with the primary antibody (Table 1) and 5% bovine serum albumin in a humidified chamber at 4 °C overnight (LIN28A, SALL4) or the slides were either blocked with 3% bovine serum albumin for 20 min (SOX9, AMH, Cx43), 30 min (SOHLH1), or with normal goat serum (1:5) at room temperature for 20 min (β-galactosidase). Then, these slides were incubated with the appropriate primary antibody in a humidified chamber at 4 °C overnight. Thereafter, all slides were exposed to a compatible horseradish peroxidase (HRP)-conjugated secondary antibody (EnVision, DAKO, Hamburg, Germany, ready to use) for 30 min (AMH, β-galactosidase, SOX9, LIN28A, SALL4) or to a biotinylated goat anti-rabbit secondary antibody (Vector Laboratories, Burlingame, CA, USA) either for 30 min (Cx43) or for 60 min (SOHLH1), followed by 30-min incubation with avidin-biotin peroxidase complex (VECTASTAIN^®^ Elite^®^ ABC Kit, Vector Laboratories, Burlingame, CA, USA) (Cx43, SOHLH1). Immunoreactivity was visualized by 3, 3′-diaminobenzidine (DAB+) substrate-chromogen. Finally, the sections were counterstained with hematoxylin, followed by dehydration and covering with Eukitt (O. Kindler GmbH, Freiburg, Germany).

### 2.5. Next-Generation Sequencing and Real-Time Reverse Transcription-PCR

#### 2.5.1. RNA Extraction

The RNeasy Lipid Tissue Mini Kit with QIAzol Lysis Reagent (Quiagen, MD, USA) was used for the extraction according to the manufacturer’s protocol. The obtained RNA was quality controlled on an Agilent 2100 Bioanalyzer system with an RNA 6000 Nano Kit (Agilent Technologies, Santa Clara, CA, USA). Only those samples with an RNA Integrity Number (RIN) value ≥ 8 were chosen for further analysis.

#### 2.5.2. mRNA Sequencing

The prepared RNA samples were quantified on a Qubit Fluorometer (Thermo Fisher Scientific, Waltham, MA, USA) and adjusted to 1000 ng. Library preparation was performed using the TruSeq Stranded mRNA Sample Preparation Kit (Illumina, San Diego, CA, USA). In total, 18 samples were run on a High Output Kit v2 (Illumina, San Diego, CA, USA) and a further six samples on a Mid Output Kit v2 on the NextSeq 500 system for 2 × 75 bp in paired-end mode. The mRNA-seq data are deposited at Sequence Read Archive (NCBI, https://www.ncbi.nlm.nih.gov/sra, project ID: PRJNA603458). The submission IDs of fastq-files, samples and genotypes used for next-generation sequencing of mRNA are shown in Table S1.

#### 2.5.3. cDNA Synthesis and Quantitative Real-Time Reverse Transcription-PCR

To validate NGS data by qRT-PCR, five candidate genes were chosen according to the following criteria: GC-specific and significantly downregulated (*p*(FDR) < 0.05) in KO mice (*Sohlh1*, *Sohlh2*), SC-specific and significantly upregulated (*p*(FDR) < 0.05) in murine KO animals (*Amh*, *Fshr*) as well as *Gja1* that codes for Cx43. *β-Actin* was selected as a housekeeping gene because no significant regulation at any age and group was observed by NGS.

Initially, obtained RNA samples with a RIN value ≥ 8 were applied to cDNA synthesis by reverse transcription using the Biometra T-Professional Thermocycler (Biometra GmbH, Göttingen, Germany) according to the manufacturer’s instructions. In order to do so, a reaction medium comprising 1x Taq DNA-Polymerase-PCR buffer (20 mM Tris HCl (pH 8.4), 50 mM KCl, 18067017, Invitrogen™, Darmstadt, Germany), 5 mM MgCl_2_ (18067017, Invitrogen™, Darmstadt, Germany), 1 mM dNTP (NU-0010-10, Eurogentec, Cologne, Germany), 2.5 µM random hexamers (N8080127, Applied Biosystems™, Darmstadt, Germany), 20 U RNAse Inhibitor (N8080119, Applied Biosystems™, Darmstadt, Germany), and 50 U moloney murine leukemia virus reverse transcriptase (M-MLV-RT) (28025013, Invitrogen™, Darmstadt, Germany) was used. Then, 200 ng of total RNA were applied to achieve a final cDNA concentration of 5 ng/µL after reverse transcription reaction. Next, the reaction volume was replenished with nuclease-free water (Fresenius Kabi AG, Bad Homburg, Germany) to a final volume of 20 µL. Subsequently, the reverse transcription reaction was performed for ten minutes at 25 °C, followed by one-hour incubation at 42 °C. Denaturation was carried out at 99 °C for five minutes, and following this the samples were flash-cooled on ice. Negative controls were conducted by renouncing reverse transcriptase and RNase inhibitor to assess DNA contamination, by omitting RNA, reverse transcriptase and RNAse inhibitor to detect possible RNA contamination as well as by excluding RNA and replacing it with Ampuwa^®^ water (Fresenius Kabi AG, Bad Homburg, Germany) as control for contamination of the reagents.

For qRT-PCR, the BioRad Real Time System CFX96 1000 Touch (BioRad, Munich, Germany) and a reaction mix, which contained 10 μL of Mesa Green PCR MasterMix Plus for SYBR^®^ Assay (Eurogentec, Cologne, Germany), and 0.2 μM concentrations of each primer (Eurofins MWG Operon, Ebersberg, Germany) for the genes of interest was used. The primers were either previously described or constructed by means of the NCBI primer designing tool [36], respectively, Primer3: WWW primer tool (http://biotools.umassmed.edu/bioapps/primer3_www.cgi) [37]; the respective sequences and accession numbers are shown in Table 2. All reactions were run in duplicates with a 20 µL volume including 1.0 µL cDNA (with a concentration of 5 ng/µL). After adding the corresponding amount of primers, the volume was filled up with nuclease-free water (Fresenius Kabi AG, Bad Homburg, Germany). For each primer pair, a negative control was implemented comprising nuclease-free water instead of cDNA. Additionally, negative controls containing RNA but no RNAse inhibitor and no reverse transcriptase as well as a negative control without RNA, RNAse inhibitor and no reverse transcriptase and one negative control comprising RNAse inhibitor and reverse transcriptase but no RNA were included as controls for the contamination of the cDNA. Cycling conditions were as follows: denaturation at 95 °C for ten minutes and then 43 cycles of following steps: denaturation at 95 °C for 15 s, annealing at 60 °C for 30 s and elongation at 72 °C for 30 s. Transcripts were visualized using Mesa Green (Eurogentec, Cologne, Germany). A subsequent melting curve was generated to verify the PCR fragments and was initiated at 55 °C to a final temperature of 95 °C with an increase of 0.5 °C every ten seconds. The relative abundance of total RNA was determined relative to the housekeeping gene (*β-Actin*), which did not differ between KO and WT littermates of all ages (*p* > 0.05). The ΔCt (delta cycle threshold) method was used to calculate the relative abundance of total RNA in the testis samples. The mean value of the Ct-merit of a specific gene was subtracted from the mean value of the housekeeping gene (*β-Actin*), and calculated by the formula 2^−ΔCt^.

#### 2.5.4. Statistical and Bioinformatics Analysis

Quality control of fastq-files was performed using FastQC (V 2.5, http://www.bioinformatics.babraham.ac.uk/projects/fastqc/). Next, fastq-files were mapped to the reference genome ‘Mouse C57BL/6NJ’ (Release 90, Ensembl) using STAR (V 0.7.13) [39]. The visualization of the KO region at the gene *Gja1* was performed using an integrative genomics viewer (IGV) [40].

The subsequent counting of mapped reads per gene was done using the RSEM software (V 1.3.0) [41]. RSEM values were further normalized by either GC or SC counts and prepared for further analysis using the VOOM transformation in the R-package limma to obtain log2 counts [42]. The final expression matrix was explored by means of principal component analysis (PCA) and hierarchical clustering to identify a potential grouping of samples. The identification of differentially expressed genes between WT and KO animals and between the three time points (days 8, 10 and 12) was again performed using the functionality of the limma package [43]. Raw *p*-values were adjusted by the method of Benjamini and Hochberg to control a false discovery rate (FDR) of 5% [44]. Enrichment of Gene Ontology (GO) terms [45] was studied using the gene-set enrichment analysis (GSEA) with the Kolmogorov–Smirnov (KS) test [46].

Cell counts are presented as mean ± standard deviation for each experimental group (*n* = 4 per age group and genotype). Next, the effect of group (KO, WT) and time (days 8, 10, 12) as well as the interaction between group and time were analysed using 2-way analysis of variance (Table 3). Subsequent comparisons between KO and WT cell counts were performed by t-tests (Figure 3). The individual time effect for the KO and WT group, respectively, was studied by 1-way analysis of variance. The level of significance was set to alpha = 0.05.

PANTHER pathway analysis was conducted using all significantly differentially expressed genes (*p*(FDR) < 0.05) for days 8, 10 and 12 p.n. and PANTHER [47,48].

Delta-Ct values (2^−ΔCt^) from qRT-PCR were compared between KO and WT samples by t-tests, and the resulting *p*-values were adjusted according the Bonferroni-Holm method.

#### 2.5.5. Further Analysis of Differentially Expressed Genes

To identify potentially interesting candidate genes (Table S2), a candidate gene list for the terms ‘infertility’, ‘idiopathic infertility’, ‘disturbed meiosis’ and ‘spermatogenic arrest’ was prepared using NCBI Gene database, for the terms ‘Sertoli cell’ and ‘Sertoli cell-specific’ generated with the UniProt Knowledgebase, and supplemented with further genes ascertained in previous studies [9,49,50] as well as with genes belonging to the GO terms which were found to be significant at all three time points (see below). Finally, only significantly expressed genes at all three time points (*p*(FDR) < 0.05) were selected as potential candidate genes and were further investigated using PANTHER [47,48,51].

## 3. Results

### 3.1. Confirmation of Cx43 Gene Loss on Protein Level

**Figure 1:**
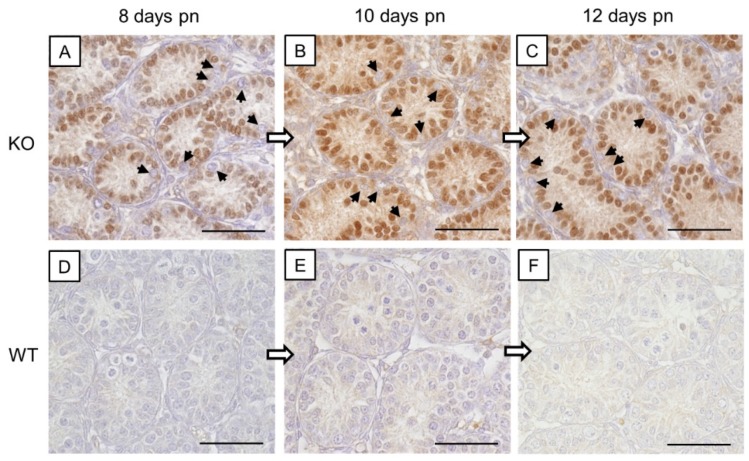
β-galactosidase immunohistochemistry. (**A**–**C**) Strong immunostaining of Sertoli cell nuclei in prepubertal SCCx43KO mice confirmed the successful deletion of the Cx43 gene. All other cells, including germ cells (arrows) were immunonegative. (**D**–**F**) No immunoreaction in Sertoli cells of WT littermates. Cx43: connexin 43, KO: knockout, pn: post-natum, SCCx43KO: Sertoli cell-specific Cx43 knockout, WT: wild type, scale bars: 50 µm, numerical aperture: 0.75.

**Figure 2:**
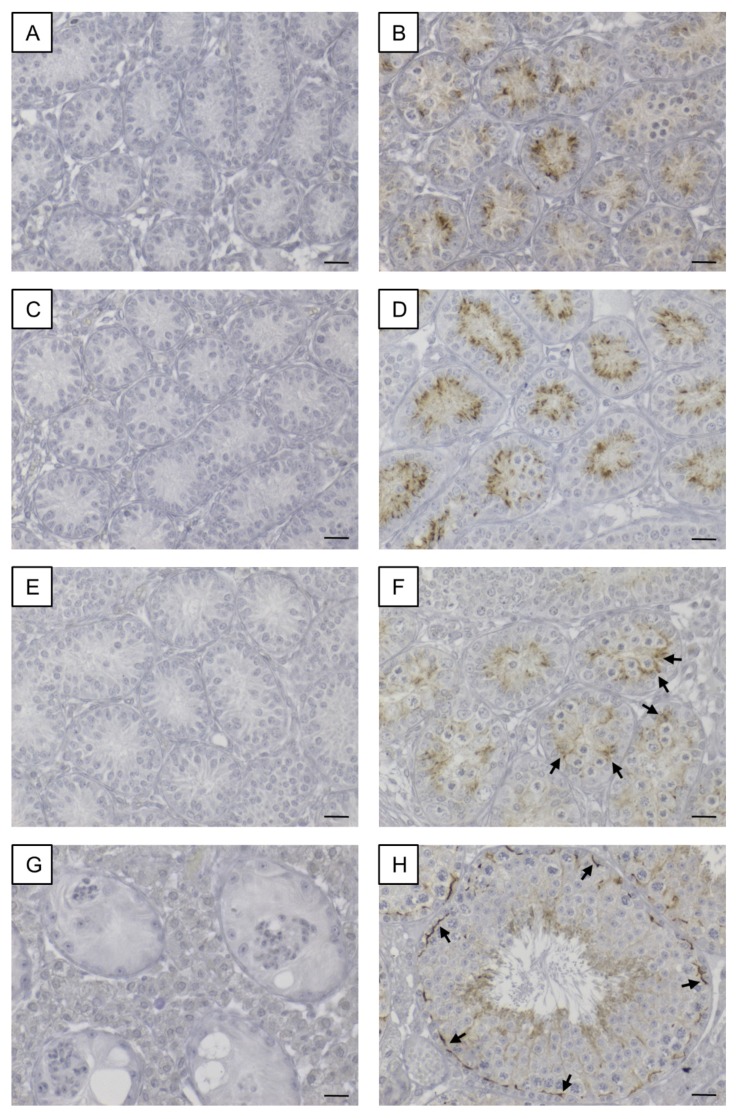
Immunohistochemical staining for Cx43. Testicular samples from SCCx43KO mice are shown on the left hand site (**A,C,E,G**) in comparison to testicular sections from WT littermates on the right hand site (**B,D,F,H**) at different ages (**A+B**: 8 days p.n., **C+D**: 10 days p.n., **E+F**: 12 days p.n. and **G+H**: adult). Within the seminiferous tubules of SCCx43KO mice, no Cx43 staining was observable in all age groups. (**B+D**) A cytoplasmic signal for Cx43 was detectable in Sertoli cells of WT animals. (**F**) Cx43 immunoreaction weakened in the Sertoli cell cytoplasm of 12-day-old WT mice and shifted in the direction of the location of the future blood-testis-barrier (arrows). (**H**) In adult WT mice, Cx43 expression was observable at the basal third of the seminiferous epithelium (arrows). Cx43: connexin 43, KO: knockout, p.n.: post-natum, SCCx43KO: Sertoli cell-specific Cx43 knockout, WT: wild type, scale bars: 20 µm, numerical aperture: 0.5.

In addition to PCR genotyping, β-galactosidase (Figure 1) and Cx43 (Figure 2) IHC were conducted to confirm the successful deletion of Cx43 protein in SCs of SCCx43KO mice.

#### 3.1.1. β-galactosidase Immunohistochemistry

A nuclear staining was solely detected in SCs in the seminiferous tubules of SCCx43KO mice (Figure 1A–C) and thus confirming a successful Cx43 gene deletion as described previously [26]. All other cells showed no immunostaining. In the seminiferous tubules of WT littermates, SCs were immunonegative (Figure 1D–F). None of the negative controls showed any immunoreaction (Figure S1A).

#### 3.1.2. Cx43 Immunohistochemistry

In SCCx43KO mice, no immunoreaction was observed in any of the seminiferous tubules, regardless of the age group (Figure 2A,C,E,G). Hence, confirmation of successful Cx43 gene loss in SCs was given again. According to Bravo-Moreno and colleagues [20] as well as Gerber and fellow workers [52], immunostaining for Cx43 was detected in seminiferous tubules of WT mice at all age groups, but with an age-dependent expression pattern (Figure 2B,D,F,H). In 8- and 10-day-old WT animals, the immunosignal appeared in the cytoplasm of SCs (Figure 2B,D). At the age of 12 days, the immunoreaction for Cx43 weakened in the cytoplasm of SCs and a shift towards the basal compartment of the seminiferous epithelium was observed (Figure 2F). Finally, in adult WT mice, as expected, Cx43 expression was detected at the location of the blood–testis barrier between adjoining SCs as well as between SCs and GCs (Figure 2H). No immunostaining was detected in the negative controls (Figure S1B).

### 3.2. Prepubertal SCCx43KO Mice Show Evident Differences in the Composition of Intratubular Cells

**Figure 3:**
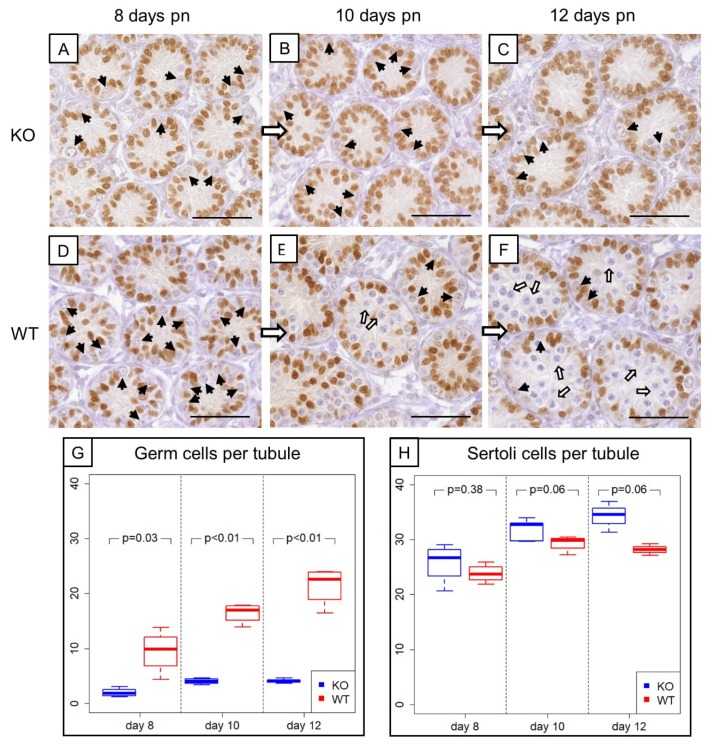
Comparison of prepubertal SCCx43KO mice with their WT littermates. (**A**–**C**) SOX9 immunohistochemistry of 8-, 10-, and 12-day-old SCCx43KO mice. Sertoli cell nuclei showed a distinct immunostaining, whereas germ cells were immunonegative (black arrows). KO mice exhibited an arrest of spermatogenesis at the level of spermatogonia. (**D**–**F**) Immunohistochemistry of SOX9 in 8-, 10-, and 12-day-old WT littermates. In WT mice, the first spermatocytes were detectable ten days pn (white arrows), whereas in KO mice only spermatogonia could be found. The difference became more pronounced with increasing age. Scale bars: 50 µm, numerical aperture 0.75. (**G**) Illustration of the average number of germ cells per seminiferous tubule of prepubertal KO and WT littermates (*n* = 4 per age group and genotype). Note already reduced germ cell numbers in 8-day-old SCCx43KO mice compared to WT littermates. (**H**) Schematic representation of mean Sertoli cell numbers per seminiferous tubule of KO and WT mice at different ages (*n* = 4 per age group and genotype). Cx43: connexin 43, KO: knockout, pn: post-natum, SCCx43KO: Sertoli cell-specific Cx43 knockout, WT: wild type.

Morphological, histological and immunohistochemical examination (Figure 3A–F) revealed evident differences in the composition of intratubular cells between 8-, 10- and 12-day-old WT and KO littermates. GC numbers per seminiferous tubule amounted on average 2 ± 0.78 GCs in 8-day-old SCCx43KO mice, 3.96 ± 0.54 GCs in 10-day-old SCCx43KO animals and 4.21 ± 0.43 GCs in 12-day-old SCCx43KO mice, whereas WT littermates had, on average, 9.49 ± 3.90 (8 days p.n.), 16.44 ± 1.80 (10 days p.n.) and 21.37 ± 3.50 GCs (12 days p.n.) per seminiferous tubule (Figure 3G).

For GCs, the main effects as well as the interaction between them were significant (Table 3). The significant interaction means that the group effect is of different size at the individual time points or, vice versa, the time effect is different for KO and WT. The results from the t-tests show that the group effect was strongest at Day 12. The individual time effect was significant for the KO as well as for the WT group (each *p* < 0.01).

For SCs in SCCx43KO mice, the following average numbers were yielded: 25.77 ± 3.63 (8 days p.n.), 31.28 ± 1.80 (10 days p.n.) and 34.16 ± 2.28 (12 days p.n.) SCs per seminiferous tubule. WT littermates showed subsequent mean numbers of SCs per seminiferous tubule: 23.8 ± 1.67 (8 days p.n.), 29.3 ± 1.43 (10 days p.n.) and 28.15 ± 0.85 (12 days p.n.) (Figure 3H).

For SCs, the group and time effects, but not the interaction between them, were significant (Table 3). Time specific comparisons did not provide further evidence for a significant difference between KO and WT samples. Thus, there is also an overall group effect. The individual time effect was significant for the KO (*p* < 0.01) as well as for the WT (*p* = 0.02) group.

In comparison to WT mice, the GC number was reduced by approximately three quarters in KO mice. Moreover, an increasing (but not significant) tendency of the SC number was observed in prepubertal SCCx43KO mice. Based on these findings, NGS data were normalized regarding GC numbers or SC numbers (see Chapter 3.3.2.).

### 3.3. Next-Generation Sequencing

#### 3.3.1. Preprocessing and Data Exploration

**Figure 4:**
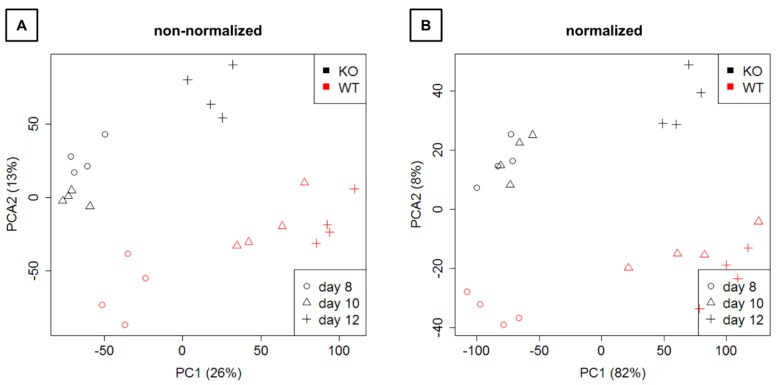
Representation of the expression data on the first two principal components. (**A**) Raw next-generation sequencing (NGS) data are shown. (**B**) Normalized data are depicted. KO: knockout, WT: wild type.

Mapping and counting resulted in expression levels for 41,097 genes in total. Exploration by PCA (Figure 4) showed a clear separation of WT and KO samples on the second principal component as well as a separation of time points on the first principal component.

**Figure 5:**
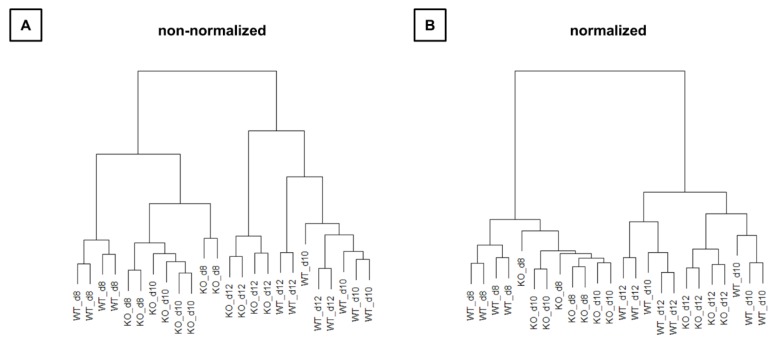
Relation of all samples to each other in hierarchical cluster trees. (**A**) Raw next-generation sequencing (NGS) data are used for representing. (**B**) NGS data are normalized. KO: knockout, WT: wild type d8: 8-day-old, d10: 10-day-old, d12: 12-day-old.

Hierarchical clustering (Figure 5) also showed that WT and KO samples were sometimes more similar than samples between time points. Thus, the time effect appears to be larger than the group effect.

#### 3.3.2. Differential Expression Analysis

The numbers of differentially expressed genes were studied in different models (Table 4). Comparisons were either performed in all samples or in subgroups divided by group (WT/KO) or time (day8, 10, 12). Analyses were either performed without cell count specific normalization or with two variants of normalization (Tables S3–S14). Note: Fc-values were calculated relating values from WT mice to values from KO mice. Hence, a positive fc-value means a downregulation of corresponding gene transcripts in the KO mice and a negative fc-value means that transcripts are upregulated in the KO animals (Tables S2–S14 and Table S21).

#### 3.3.3. Gene Ontology Analysis by GSEA

In total, 8904 GO terms were related to the genes of the transcriptome expression matrix. GO term enrichment was either studied for the comparison of KO versus WT based on all samples or based on time (Day 8, 10, 12). Due to the resampling strategy of GSEA and the use of the nonparametric KS test, the resulting *p*-values had only a precision of three digits. With this limitation, 158 GO terms yielded a *p*-value of 0 for the comparison of KO versus WT based on all samples (Table S15). The ‘GO:0007141 male meiosis I’ was found among others. The time specific comparison of KO versus WT samples revealed 180 GO terms at day8, 47 GO terms at Day 10 and 246 GO terms at Day 12 with a *p*-value of 0 (Tables S16–S18). Ten GO terms with a *p*-value of 0 were found at all three time points. Table 5 lists these ten GO terms including their corresponding genes.

#### 3.3.4. PANTHER Pathway Analysis of Significantly differentially Expressed Genes

In order to gain a general overview of involved pathways, PANTHER pathway analysis was performed with all significantly differentially expressed genes in 8-, 10- and 12-day-old SCCx43KO mice (*p*(FDR) < 0.05). In total, 133 pathways were identified (Table S19). Among these, the ‘gonadotropin-releasing hormone receptor pathway’ showed the most gene hits, followed by ‘Wnt signaling pathway’, ‘CCKR signaling’ and ‘inflammation mediated by chemokine and cytokine signaling pathway’.

#### 3.3.5. Further Characterization of Differentially Expressed Genes

**Figure 6:**
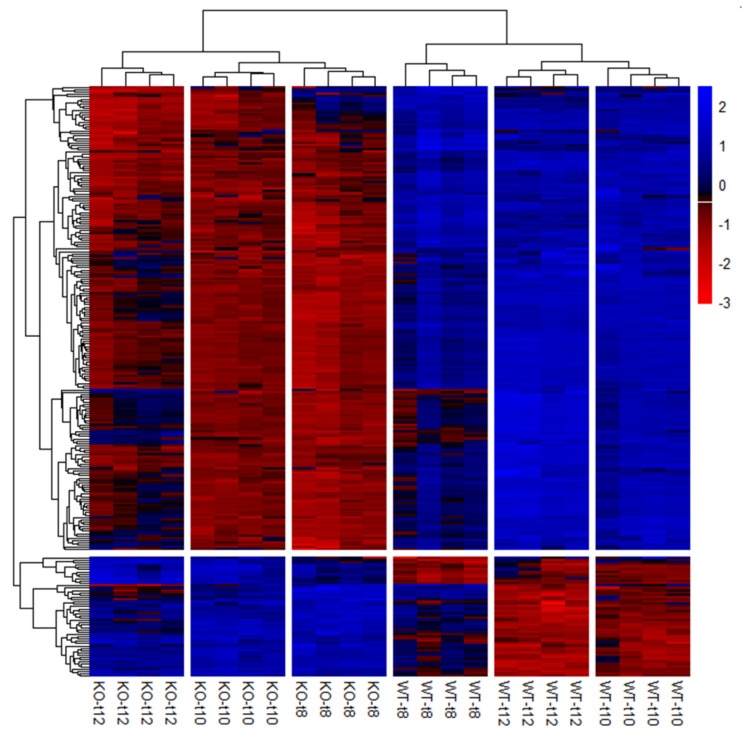
Hierarchical clustering and heat map of the 254 significantly regulated candidate genes. Each column represents a sample of SCCx43KO mice or their WT littermates at different ages (*n* = 4 per genotype and age group, t8: 8-day-old, t10: 10-day-old, t12: 12-day-old). Genes are depicted in rows. Red indicates downregulation and blue indicates upregulation. KO: knockout, SCCx43KO: Sertoli cell-specific Cx43 knockout, WT: wild type.

Preparation of the candidate gene list (Table S2) revealed 254 potential candidate genes that were significantly differentially expressed (*p*(FDR) < 0.05) in the SCCx43KO animals in all three age groups (Figure 6).

In order to identify the most significantly differentially expressed candidate genes in 10-day-old animals, the candidate gene list was sorted by *p*(FDR)-values and a literature review was conducted for the first 15 genes (Table 6). Noteworthy, all of these 15 genes were significantly downregulated in the 8-, 10- and 12-day-old SCCx43KO mice. 

**Figure 7:**
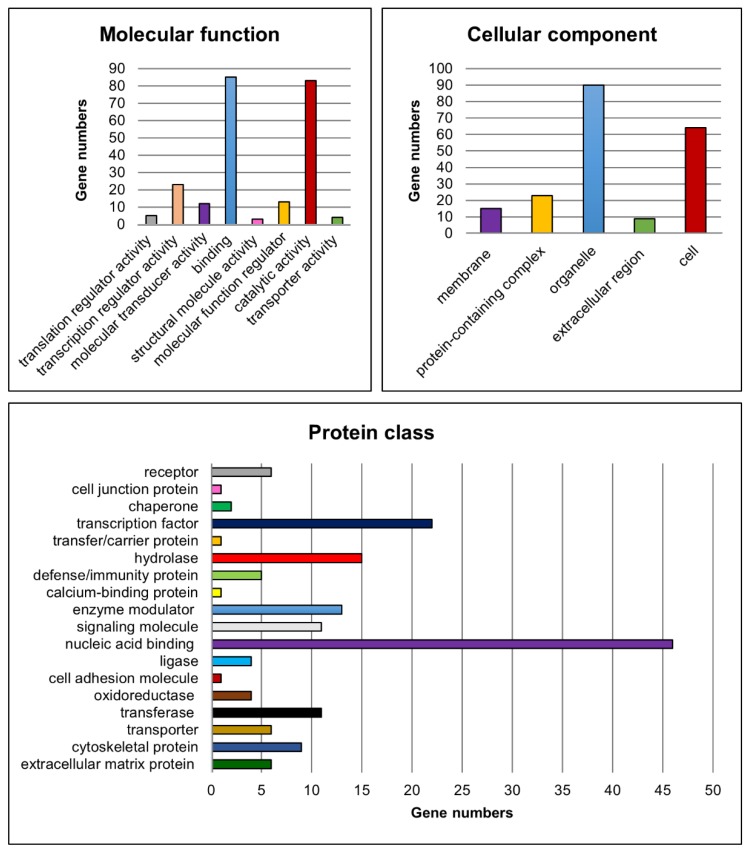
Functional classification of potential candidate genes. Charts showing numbers of potential candidate genes related to the corresponding gene ontology categories for the terms molecular function, cellular component and protein class.

Moreover, functional classification of the candidate genes was performed for the molecular function, cellular component, protein class (Figure 7), biological process and pathways to assign the biological information of the transcriptome analyses using PANTHER [47,48,51].

For the molecular function category, genes implicated in ‘binding’ (e.g., *Lhx8*, *Rb1* and *Ovol1*) and ‘catalytic activity’ (e.g., *Fshr*, *Kit* and *Cyp26b1*) were found to be most abundant amongst the significantly differentially expressed candidate genes. Genes such as *Sohlh1*, *Sohlh2*, *Ovol1*, *Sox3* and *Gata4* were related to ‘transcription regulator activity’. With regard to the cellular component, genes belonging to the GO categories ‘organelle’ (e.g., *Sall4*, *Dmrtc2* and *Rec8*) and ‘cell’ (e.g., *Dazl*, *Inha* and *Inhbb*) were mostly represented. Furthermore, the GO categories ‘nucleic acid binding’ (e.g., *Lin28a*, *Rb1* and *Tdrd1*), ‘transcription factor’ (e.g., *Grhl1*, *Otx1* and *Taf7l*) and ‘hydrolase’ (e.g., *Usp26*, *Mov10l1* and *Ctsl*) were the most represented terms related to the protein class. With respect to the already known functions of the gap junction protein Cx43, genes belonging to the cellular component category ‘membrane’ (e.g., *Insr*, *Fgfr3*, *Tyro3*) and to the protein classes ‘cell junction protein’ (e.g., *Ocln*), ‘cell adhesion molecule’ (e.g., *Sdc1*) as well as ‘cytoskeletal protein’ (e.g., *Ttll5*, *Spag4* and *Fignl1*) might also be of interest for investigating possible molecular mechanisms leading to observed spermatogenic arrest at the spermatogonial level in SCCx43KO mice.

Regarding the biological process, especially genes involved in ‘cellular process’, ‘biological regulation’ and ‘metabolic process’ were found to be highly represented. For this survey, all GO categories and associated genes are summarized in Table 7. Finally, the PANTHER pathway analysis yielded 46 pathways in relation to the candidate genes (Table S20). ‘Gonadotropin-releasing hormone receptor pathway’, ‘p53 pathway’ and ‘CCKR signaling map’ were the three categories with the most gene hits but the majority of PANTHER pathways were associated with four or fewer of the candidate genes (Figure S2).

### 3.4. Confirmation of NGS Candidate Genes by qRT-PCR

**Figure 8:**
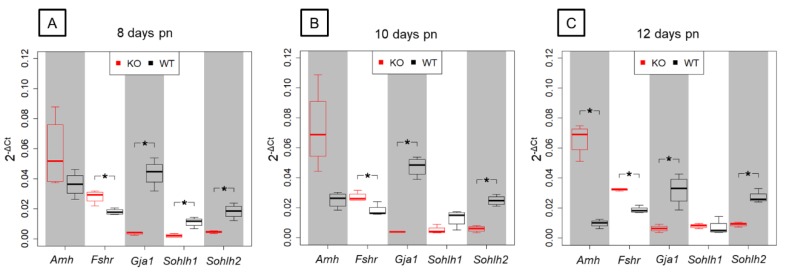
Relative gene expression in prepubertal SCCx43KO mice and their WT littermates. Box plots showing 2^−ΔCt^-values of relative gene expression in KO and WT littermates for the candidate genes *Amh*, *Fshr*, *Gja1*, *Sohlh1* and *Sohlh2* between KO and WT littermates in three age groups (*n* = 4 per genotype and age group, *p*(FWER) < 0.05). (**A**) In 8-day-old SCCx43KO, *Fshr*, *Gja1*, *Sohlh1* and *Sohlh2* are significantly, differentially expressed. (**B**) *Fshr*, *Gja1* and *Sohlh2* are significantly, differentially regulated in 10-day-old SCCx43KO mice. (**C**) A significant, differential gene expression is detectable for *Amh*, *Fshr*, *Gja1* and *Sohlh2* in 12-day-old SCCx43KO mice. qRT-PCR data correlate with NGS data. RNA expression level is normalized to the expression level of the housekeeping gene *β-Actin*. KO: knockout, NGS: next-generation sequencing, pn: post-natum, qRT-PCR: quantitative real-time reverse transcription-PCR, SCCx43KO: Sertoli cell-specific Cx43 knockout, WT: wild type.

In order to validate results obtained by NGS, five candidate genes that were found to be differentially expressed in the prepubertal SCCx43KO animals and related to spermatogenesis were selected for qRT-PCR analysis (Figure 8).

Among the chosen genes, SC-specific *Amh* and *Fshr* were found to be significantly upregulated by NGS analysis showing a decreasing *p*(FDR)-value with increasing age. Relative gene expression levels observed by qRT-PCR were higher in the SCCx43KO animals compared with their WT littermates for *Amh* and *Fshr* but differences in *Amh* gene expression were only significant in the 12-day-old age group. Furthermore, NGS revealed a significant downregulation of *Gja1*, *Sohlh1* and *Sohlh2* in SCCx43KO mice in each age group. For *Gja1*, *Sohlh1* and *Sohlh2*, the relative abundance of total RNA was found to be lower in SCCx43KO animals in comparison with WT mice by qRT-PCR. Differences in *Gja1* and *Sohlh2* gene expression were significant in all three age groups, whereas *Sohlh1* expression was significantly regulated in the 8-day-old age group. In general, qRT-PCR data were found to correlate with results received by NGS.

### 3.5. Confirmation of Candidate Genes at Protein Level by Immunohistochemistry

#### 3.5.1. AMH

*Amh* gene, coding for the anti-Müllerian-Hormone, a factor synthesized by prepubertal SCs, was selected for validation because it was among the significantly and differentially upregulated genes in SCCx43KO mice at all three age groups and it is known as marker of immature SCs (reviewed in [83,84], see also [35]).

**Figure 9:**
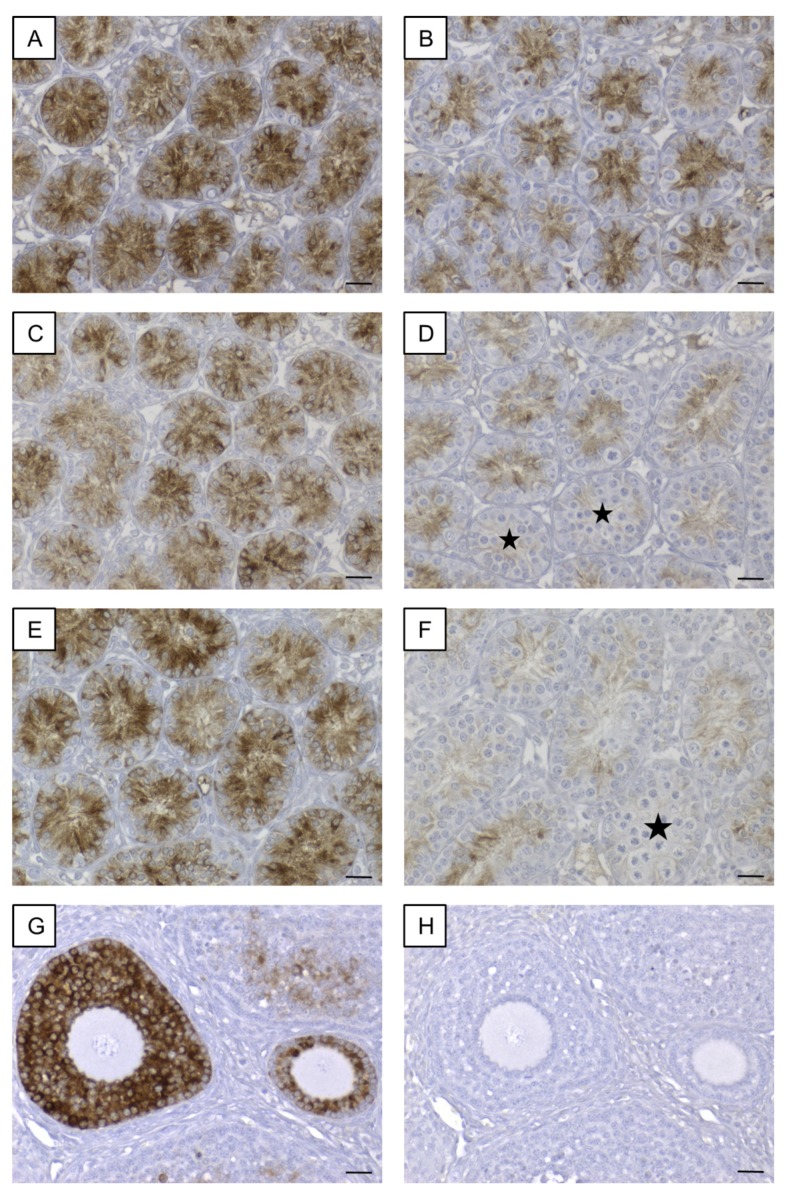
Immunohistochemical detection of AMH. (**A**) Strong immunoreaction in Sertoli cell cytoplasm of 8-day-old SCCx43KO mice. (**B**) Distinct, cytoplasmic immunosignal in Sertoli cells of 8-day-old WT littermates. (**C**) Sertoli cell cytoplasm of 10-day-old SCCx43KO animals still showed a strong immunostaining. (**D**) Immunoreaction weakened in Sertoli cells of 10-day-old WT littermates. First seminiferous tubules were almost immunonegative (asterisks) concomitant with the first appearance of spermatocytes. (**E**) In 12-day-old SCCx43KO mice, a strong, cytoplasmic immunosignal was still observed in Sertoli cells. (**F**) Faint or almost no (asterisk) immunostaining of Sertoli cell cytoplasm of WT animals. (**G**) Mouse ovary tissue as exemplary positive control. (**H**) Exemplary negative control of mouse ovary tissue. AMH: anti-Müllerian-Hormone, Cx43: connexin 43, SCCx43KO: Sertoli cell-specific Cx43 knockout, WT: wild type, scale bars: 20 µm, numerical aperture: 0.5.

Immunohistochemical staining for AMH revealed a strong cytoplasmic signal in SCs of seminiferous tubules of 8-day-old SCCx43KO mice (Figure 9A). In WT littermates (8 days p.n.), immunostaining was observed at the same localization but the immunoreaction was not that strong (Figure 9B). SC cytoplasm in all seminiferous tubules of 10-day-old SCCx43KO mice also showed a distinct immunosignal (Figure 9C), whereas in the 10-day-old WT littermates (Figure 9D), the immunostaining weakened and single seminiferous tubules were found to be almost immunonegative for AMH (asterisks). In addition to SCs, these seminiferous tubules comprised first primary spermatocytes while immunopositive tubules so far only contained spermatogonia. In 12-day-old SCCx43KO animals, the cytoplasm of SCs was still found to be clearly immunopositive (Figure 9E) but in seminiferous tubules of WT mice, a faint or almost no (asterisk) immunosignal was observed (Figure 9F). The manufacturer recommends using mouse ovary tissue as a control tissue for AMH IHC. Thus, mouse ovary tissue was added as a positive control to ensure a correct immunohistochemical staining against AMH (Figure 9G). As negative controls, both mouse ovary (Figure 9H) as well as testicular samples (Figure S1C) were used. None of the negative controls showed any immunostaining (Figure S1C).

#### 3.5.2. LIN28A and SALL4

**Figure 10:**
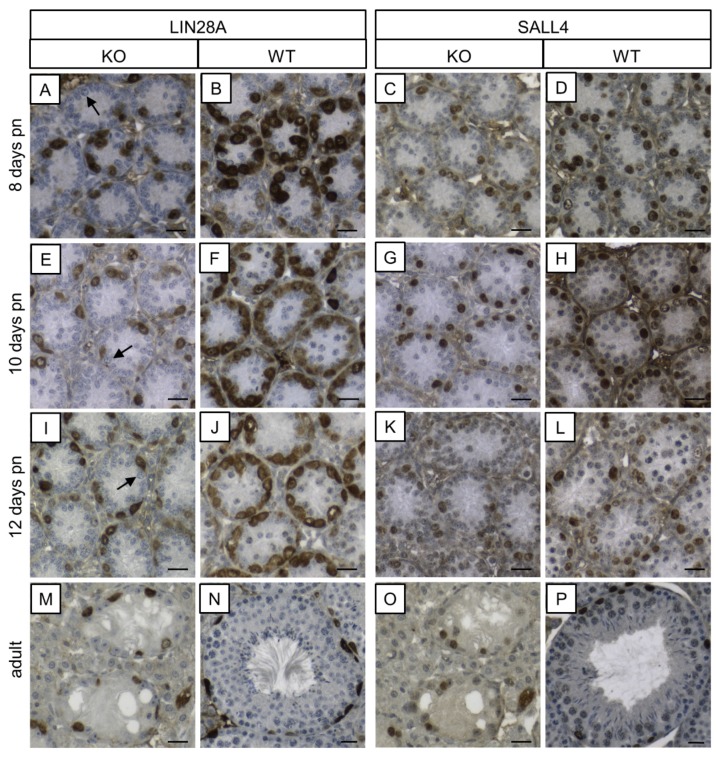
Immunohistochemical detection of LIN28A and SALL4 in SCCx43KO and WT mice. (**A,E,I**) In seminiferous tubules of prepubertal KO mice, almost all spermatogonia are immunopositive for LIN28A, except for a few single ones (arrows). (**M**) In adult KO mice, seminiferous tubules show a spermatogenic arrest at the spermatogonial level. LIN28A immunolabeling is observable in remaining spermatogonia. (**B,F,J,N**) Seminiferous tubules of WT mice at different ages show an immunostaining according to the age. (**C,G,K,O**) In prepubertal and adult KO mice, spermatogonia are immunopositive for SALL4. (**D,H,L,P**) SALL4 immunoreactivity is detectable in spermatogonia in a typical pattern according to the age in the prepubertal and adult WT mice. KO: knockout, pn: post-natum, SCCx43KO: Sertoli cell-specific Cx43 knockout, WT: wild type, scale bars: 20 µm, numerical aperture: 0.5.

In order to examine the protein levels of two genes that are markers for undifferentiated spermatogonia (reviewed in [85]), LIN28A and SALL4 IHC were performed (Figure 10). Almost all spermatogonia were found to be immunolabeled for LIN28A in the prepubertal KO mice with the exception of a few single GCs. In adult SCCx43KO mice, seminiferous tubules showed SCO tubules or an arrested spermatogenesis at the spermatogonial level. The remaining spermatogonia were immunopositive for LIN28A. Immunoreactivity for LIN28A was detectable in GCs in prepubertal and adult WT mice. Moreover, immunopositive cells for SALL4 were observed in both genotypes and at all ages. Except for the difference regarding GC numbers, no alterations in the staining patterns were detected between SCCx43KO and WT animals.

#### 3.5.3. SOHLH1

IHC was conducted to examine the protein expression level of a further gene, which is GC-specific [50] and which was significantly differentially expressed (downregulated) at the mRNA level in KO mice in comparison with WT littermates.

**Figure 11:**
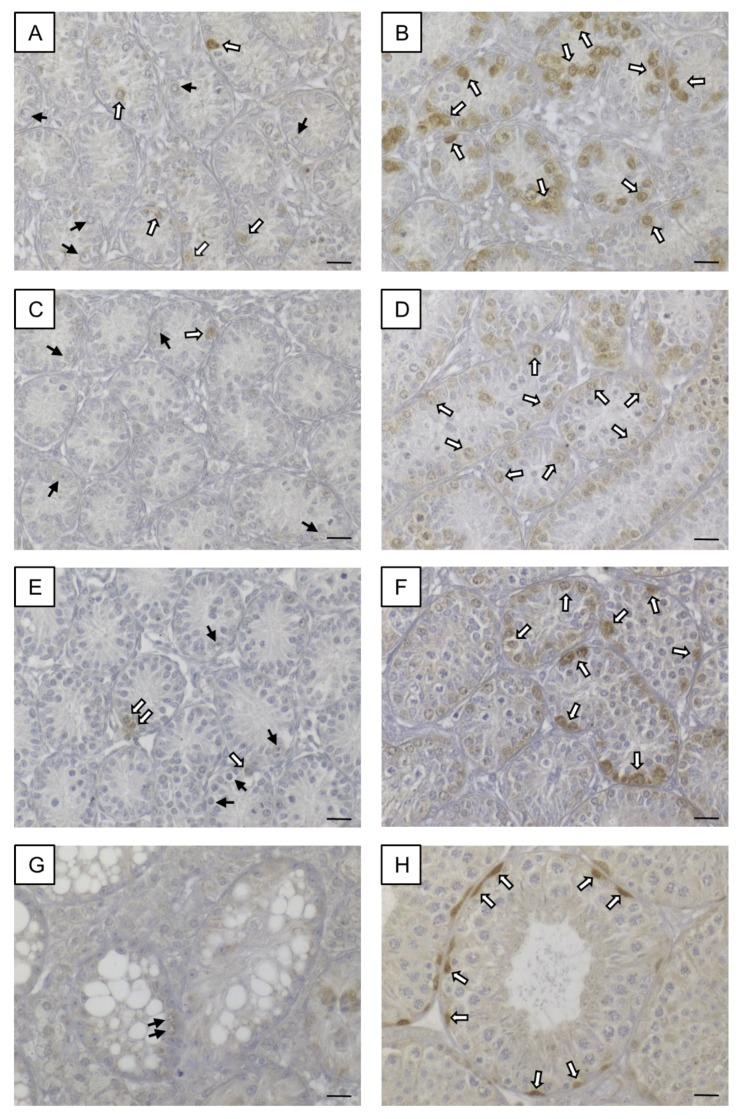
SOHLH1 immunohistochemistry. (**A**) Seminiferous tubules of 8-day-old SCCx43KO mice contained some immunopositive spermatogonia (white arrows) but also immunonegative spermatogonia (black arrows). (**B**) Strong immunosignal in almost all spermatogonia in seminiferous tubules of 8-day-old WT littermates (white arrows). (**C,E**) In seminiferous tubules of 10- and 12-day-old SCCx43KO animals, single immunopositive spermatogonia were observed (white arrows) but most spermatogonia showed no immunostaining (black arrows). (**D,F**) Distinct immunoreaction was found in spermatogonia of 10- and 12-day-old WT mice. (**G**) No immunosignal was observed in germ cells (black arrows) of adult SCCx43KO animals. (**H**) A spermatogonial subpopulation showed strong immunostaining (white arrows) in adult WT mice. KO: knockout, SCCx43KO: Sertoli cell-specific Cx43 knockout, WT: wild type, scale bars: 20 µm, numerical aperture: 0.5.

In seminiferous tubules of KO mice at all age groups, SOHLH1 protein was rarely detectable in some spermatogonia but the vast majority of this GC population did not show any immunoreaction (Figure 11A,C,E), whereas in seminiferous tubules of WT littermates at all age groups, a strong immunostaining was observable in spermatogonia (Figure 11B,D,F). In addition, adult SCCx43KO mice (Figure 11G) and adult WT animals (Figure 11H) were immunohistochemically examined. In adult seminiferous tubules of SCCx43KO mice, no immunosignal in GCs was detected. Immunopositive spermatogonia were observed in seminiferous tubules of adult WT mice according to previous findings [50]. SCs, Leydig cells and peritubular myoid cells appeared as constantly immunonegative in both genotypes. Negative controls showed no immunoreaction (Figure S1D).

## 4. Discussion

Spermatogenesis is a highly complex process by which some spermatogonial stem cells undergo phases of mitotic proliferation and differentiation, meiotic division as well as transformation to give rise to mature sperm [86,87]. SCs assume the role of ‘nurse cells’, for example providing nutrients required by GCs and coordinating intraepithelial movement; in brief, SCs support spermatogenesis [88]. Thus, to enable successful spermatogenesis, a functional intercellular communication between SCs and GCs is necessary. In mammalians, intercellular communication either takes place through tubular structures (tunneling nanotubes (TNTs)) joining adjacent cells, through secreted molecules, which can be soluble or enclosed in vesicles, as well as through intercellular channels that connect the cytoplasm of neighboring cells [89]. Recently, Cx43 has been shown to be involved in all these types of intercellular communication (reviewed in [89]).

Hence, the current study further investigates the intriguing functions of Cx43 in SCs for spermatogenesis and SC maturation. As it has recently been reported, the expression pattern of Cx43 is altered in different forms of human testicular disorders as well as in preinvasive GCNIS and TGCTs developing therefrom [6,7,8,27,28,29,90]. Establishing a SCCx43KO mouse model provided a good opportunity for studying underlying molecular mechanisms leading to spermatogenic impairment [25,26]. A previous microarray-analysis has already shown that the gene expression of many genes is affected in 8-day-old mice by the deletion of Cx43 [9]. In order to examine the effects of Cx43 loss over a greater period of time and more precisely, supplementary to 8-day-old animals, 10- and 12-day-old mice were used.

In addition to PCR genotyping and morphological evaluation by H&E staining, IHC for β-galactosidase and Cx43 were conducted to determine and confirm murine geno-/phenotypes. Observed β-galactosidase immunostaining of SC nuclei in KO mice of the different age groups (Figure 1A–C) again confirmed successful deletion of Cx43 gene because transcription of the *LacZ*-reporter gene is only possible when the previous gene sequence (Cx43 coding region) is absent [91,92]. These results are in accordance with observations of Cx43 IHC (Figure 2). Thereby, Cx43 expression was not detectable in any KO mice of each age group, again confirming the successful lack of Cx43 in SCs.

Furthermore, to clearly distinguish between GCs and SCs in the present study, SOX9 IHC was used to mark SC nuclei and then to determine intratubular cell numbers revealing reduced GC numbers in KO mice pursuant to recent findings [9,93]. However, in contrast to Giese et al. [9], increased SC numbers per seminiferous tubule have already been detected in 8-day-old KO mice and become more evident in 10- and 12-day-old KO animals (Figure 3), implying an enhanced SC proliferation already at this stage. Thus, these present findings appear to be a reasonable explanation for finally increased SC numbers in adult KO mice [26] as well as for the observed adult SC proliferation [25].

In order to analyze and compare the testicular transcriptome of prepubertal KO mice and their WT littermates, NGS was performed, because it has been shown to be a more precise method than others [94]. As suspected, transcriptome analysis revealed much more significantly and differentially expressed genes in 8-day-old KO mice in comparison with the previous microarray study (after GC-specific normalization: 4253 versus 658 genes) [9]. However, former candidate genes, such as *Stra8* and *Dazl*, were also found to be significantly regulated, confirming previous findings [9]. The greatest changes in differentially expressed genes (34,325 genes after GC-specific normalization) were detected by comparison of the 10-day-old KO mice with their WT littermates. These findings are according to the observed morphologic differences in the composition of intratubular cell populations between KO and WT mice. As described by Bellve in 1977 [32], spermatocytes were first found in 10-day-old WT mice, while in their KO littermates, only spermatogonia were present. By comparing the 10-day-old WT group with the 8-day-old WT mice, 32,042 genes were found to be significantly regulated (after GC-specific normalization), whereas the comparison of 10-day-old KO mice with the 8-day-old KO group yielded 532 significantly differentially expressed genes indicating big differences in gene expression in aging WT animals in relation to the KO mice and an impairment or delay on the KO side. These findings appear to be consistent with the occurring morphologic differences in WT mice (10 days p.n. versus 8 days p.n.) and the minor changes in KO littermates. Moreover, with advancing age, the numbers of significantly differentially expressed genes are lower in WT mice as in KO mice (12 days p.n. versus 8 days p.n.). Rising SC numbers in KO mice may explain these results, but further research is required to investigate the gene expression in an age-dependent manner.

Based on the generated NGS data, different hypotheses are conceivable to explain the development of arrested spermatogenesis at the spermatogonial level and infertility in SCCx43KO mice: (1) retinoic acid (RA) signaling might be altered; (2) maturation of SCs might be affected by the Cx43 deletion, leading to a sustained ‘immature’ cell fate which prevents SCs from completing functions required for successful spermatogenesis; (3) spermatogonial stem cell self-renewal might be disturbed; (4) differentiation of spermatogonia might be altered; (5) initiation of meiotic prophase I might be impaired; and (6) cell-to-cell communication between SCs and GCs might be interrupted.

In order to gain an overview of gene sets affected by the Cx43 deletion, GO enrichment analysis was performed, highlighting primarily processes related to meiosis. Thus, this seemed to be consistent with the observed phenotypes of KO animals that show an arrested spermatogenesis at the level of spermatogonia, and additionally, supported the valid experimental design.

Meiosis describes the production of haploid cells from diploid progenitor cells, and formation of preleptotene spermatocytes is considered as the initiation of this process [54]. In this case, the transition of undifferentiated A-spermatogonia into A1 differentiating spermatogonia could be regarded as commitment to meiosis [54]. For the progression into differentiating A1-spermatogonia, at least RA is required and subsequently STRA8 protein has been shown to be essential for inducing meiosis [54,62,63,95].

Hence, one hypothesis to explain the observed testicular phenotype in SCCx43KO mice might be that the deletion of Cx43 in SCs alters RA signaling, whereby the initiation of meiosis is disturbed and consequently results in arrested spermatogenesis at the spermatogonial level. Genes involved in RA signaling were found to be significantly differentially expressed in the prepubertal SCCx43KO mice (e.g., *Stra6*, *Stra8*, *Rarg*, *Cyp26b1 Crabp1*, *Crabp2 Aldh1a1* and *Aldh1a3*).

In the seminiferous epithelium, both SCs and spermatogonia have RA receptors (RARs) and enzymes that are required for the oxidation of retinol to RA [54]. Noteworthy, in the testis, the only putative receptor that is able to take up the circulating form of retinol (ROL-RBP4-TTR) appears to be STRA6, which is expressed primarily in SCs (reviewed in [54]). Thus, SCs are assumed as source of RA and then undifferentiated GCs represent the target cells [54]. However, it has been suggested that preleptotene spermatocytes may be another source of retinoic acid at the time of meiotic initiation [96]. Meanwhile, SC-derived RA has been shown to be pivotal for the initial differentiation of spermatogonia and the onset of meiosis in juvenile mice but not necessary after the first wave of spermatogonial differentiation [96,97]. RA promotes the spermatogenic process, on the one hand by promoting the expression of spermatogonial stem cells to differentiating spermatogonia through activation of KIT expression, and on the other hand by promoting the expression of STRA8 [95,98], thus inducing meiosis [63].

*Stra8*/STRA8 expression has previously been examined in prepubertal and adult SCCx43KO mice in comparison with their WT littermates and found to be altered or absent [9,93]. NGS data of the present study revealed a significant downregulation of *Stra8* in 8-, 10-, and 12-day-old SCCx43KO mice compared to their WT littermates. Additionally, *Stra6*, *Aldh1a1*, *Aldh1a3* and *Cyp26b1* were significantly upregulated, while *Rarg*, *Crabp1*, *Crabp2* were downregulated. These findings might indicate alterations in the RA signaling pathway caused by the absence of Cx43. In particular, it is thinkable that altered expression of cellular retinol-binding proteins leads to problems regarding storage, cell uptake and transportation in the prepubertal SCCx43KO mice. Furthermore, significant upregulation of *Cyp26b1* may imply an induced degradation of RA and in the following missing stimulation of RA-target genes, for example SOHLH1 and STRA8, which in turn may lead to altered spermatogonial differentiation and impaired meiosis in the mutant mice. Upregulation of *Stra6*, *Aldh1a1* and *Aldh1a3* may then be the response of reduced RA levels.

In addition, *Rarg* (encoding the RARγ receptor) was found to be significantly downregulated in the 8-, 10- and 12-day-old SCCx43KO mice. Recently, it has been suggested that this receptor is pivotal for spermatogonial differentiation after the first wave of spermatogenesis; as in mutant mice, the transition of A_al_ into A_1_ spermatogonia has been shown to be impaired [99]. During the first wave of spermatogenesis, meiotic cells appear but adult mice lacking RARγ show an arrested spermatogenesis at the spermatogonial level or SC-only tubules [99] comparable to the adult phenotype of the SCCx43KO mice [25,26]. Therefore, alterations of *Rarg* gene expression might also contribute to the disturbed spermatogenesis in SCCx43KO mice.

So far, various studies of different tissues, including testicular tissue, demonstrated that RA also stimulates the expression of Cx43 [17,100,101,102,103,104]. The other way round, it has been shown that defective RA signaling leads to altered gap-junction-based cell coupling as well as to alterations in tight junction formation and increased blood–testis barrier permeability [105,106,107]. However, both the exact mechanism and the link to Cx43 still remain unclear and further research needs to be performed to address this issue.

Moreover, PANTHER pathway analysis identified 133 pathways for all significantly differentially expressed genes and 46 pathways for the compiled candidate gene list that are affected by Cx43 loss. Interestingly, the pathways with the most gene hits were identical. Hence, this underlines the importance of Cx43 for these pathways and direct cell-cell communication. Furthermore, it strengthens the value of the established candidate gene list for further evaluation and for identifying possible candidate genes for future examination of corresponding human deficiencies.

For example, the ‘gonadotropin-releasing hormone receptor pathway’ showed the most gene hits (e.g., *Amh*, *Gata4*, *Inha*, and *Inhbb*). Gonadotropin-releasing hormone (GnRH) receptor signaling plays a key role in the reproduction of vertebrates [108]. Pulsatile GnRH release from the hypothalamus activates GnRH receptors in gonadotrophs of the pituitary gland leading to the secretion of e.g., follicle-stimulating hormone (FSH) [108,109]. FSH binds receptors on SCs, stimulating various cellular mechanisms, e.g., protein synthesis by SCs and SC growth [109]. In addition to others, inhibin is produced and secreted by SCs, which negatively influences hormone release in the hypothalamus and pituitary gland [84].

Another hypothesis to explain the testicular phenotype of SCCx43KO mice might be that the loss of Cx43 alters SC proliferation/maturation in the mutant mice, resulting in arrested spermatogenesis at the spermatogonial level due to asynchronous differentiation of both SCs and GCs. This assumption is supported by the observation of SCs possessing immature characteristics in mice and men with impaired spermatogenesis [35,110,111,112,113,114,115].

*Inha* (coding for inhibin A and inhibin B), *Inhbb* (coding for activin B) and *Amh* are all known to be crucial for functional regulation of reproduction and development [116]. Especially during the onset of the first wave of spermatogenesis, the modulation of synthesis and action of inhibins and activins appear to be important [117]. In contrast to activin A, which has been shown to be highly concentrated in testes during SC proliferation in newborn mice and whose concentration decreases in aging mice to a low level in adult animals, concomitantly with decreasing SC proliferation, less is known about activin B [116,117]. NGS data revealed a significant upregulation of *Inhbb* in SCCx43KO mice in all three age groups in the present study. Considering the increase in SC numbers in SCCx43KO compared to their WT littermates, these results might indicate that activin B is also involved in SC proliferation.

Interestingly, *Inha* was also found to be significantly upregulated in SCCx43KO mice at each investigated age. Physiologically, *Inha* mRNA levels progressively decrease to 30% of the 0 days p.n. value within six, ten and 12 days p.n. [117]. Total testicular levels of inhibin have been shown to increase from 0 days p.n., peaking at four days p.n., showing a plateau to Day 16 p.n., and subsequently decreasing, thereby reflecting a period of SC proliferation followed by achieving a stable, non-mitotic SC population [117]. Taking these findings [117] into account, the observed *Inha* upregulation in SCCx43KO mice compared to their WT littermates might indicate a prolonged and enhanced period of SC proliferation in the KO animals.

Additionally, *Amh* was found to be significantly upregulated in SCCx43KO mice compared to their WT littermates in all three age groups by NGS. Using qRT-PCR, this upregulation has been confirmed (Figure 8). However, *Amh* gene expression was not yet significantly altered in 8- and 10-day-old KO mice (performing qRT-PCR) underlining the precise measuring of transcript levels by NGS. Consistent with these data, an altered AMH protein expression pattern was observed in KO mice (Figure 9) according to Weider et al. [35]. AMH is the first protein secreted by fetal SCs and its expression is maintained until puberty [118,119]. In particular, androgens are known to regulate AMH expression by SCs [119,120], and also maturation of GCs negatively affects AMH production, whereas AMH secretion is stimulated by FSH in the absence of the inhibiting effect of androgens [121]. Prolonged AMH expression in the KO mice suggests that SCs are in a sustained ‘immature’ state of proliferation and therefore unable to fulfil nurse-like and scaffolding roles required for successful spermatogenesis. This suggestion is supported by the upregulation of further genes, such as the above-mentioned genes (*Inha* and *Inhbb*) as well as *Thra*, *Krt18* (markers for immature SCs [113,122]), *Fshr*, *Fgf9* and *Kitl* (also known as SCF).

FGF9 is a meiotic inhibiting substance normally produced by Sertoli cells to prevent gonocytes from entering meiosis [123]. SCs also produce KIT ligand (KITL), which has been reported to stimulate KIT expression of differentiating spermatogonia thereby promoting spermatogonial differentiation and gene expression of genes that are specific for early meiotic phases [123]. In the SCCx43KO mice, *Fgf9* (ten and twelve days p.n.) and *Kitl* (eight, ten and twelve days p.n.) were found to be significantly upregulated while *Kit* (also known as *c-kit*) was significantly downregulated in 8-, 10- and 12-day-old animals. Hence, imbalances of SC signaling might be traced back to altered SC maturation in consequence of Cx43 absence and, together with the detected alterations of *Kit* expression, these might be involved in the development of the observed testicular phenotype in SCCx43KO animals.

Interestingly, in this case, *Gata4* was significantly upregulated in the prepubertal SCCx43KO mice, too. SCs express GATA4 from the onset of their differentiation through to adulthood (reviewed in [124]). Among others, *Dmrt1*, *Inha*, *Inhbb* and *Amh* have been identified as putative target genes for GATA4 in the testis (reviewed in [124]). Thus, upregulated *Gata4* gene expression in the SCCx43KO animals may contribute to the differential gene expression of *Amh, Inha* and *Inhbb* in the transgenic mice.

Furthermore, *Dmrt1* gene knockout leads to disorganized seminiferous tubules, pre-meiotic GC death and abundance of immature SCs [125]. In 7-day-old *Dmrt1* KO mice, *Gata1* mRNA levels have been found reduced, *Gata4* and *Krt18* mRNA levels increased [126]. In this context, it has been suggested that DMRT1 plays an important role for SC maturation and its absence prevents cessation of SC proliferation as consequence of failed terminal differentiation [127]. As *Dmrt1* KO mice have a similar testicular phenotype and significantly, downregulated *Dmrt1* transcripts were observed in the prepubertal SCCx43KO mice, it seems likely that loss of Cx43 may alter SC proliferation via a same/similar regulatory mechanism resulting in impaired spermatogenesis.

Moreover, the candidate gene list drew attention to *Rb1* (also known as *Rb*) that encodes the retinoblastoma-associated protein, a key regulator of cell cycle entry as well as cellular division, and a tumor suppressor [128,129]. It was generally assumed that adult SCs are terminally differentiated. Recent studies, however, supposed that SCs are rather in a state of continuous cell cycle repression and that the non-proliferative state of adult SCs is maintained by RB1 [129,130,131]. Rotgers and colleagues have shown that repression of the transcription factor E2F3 by RB1 is decisive for the maintenance of cell cycle quiescence in adult SCs [122]. Loss of *Rb1* leads to impaired cell cycle control in the form of cell cycle re-entry mediated by E2F3 and hence to increased SC proliferation and loss of SC function [122,131]. Furthermore, *follistatin (Fst)* expression was found to be increased in the absence of *Rb1*, which seems to be due to a potentially E2F3-mediated mechanism [122].

With regard to the SCCx43KO mice, NGS data revealed a significant downregulation of *Rb1* in all age groups, no changes in *E2F3* gene expression and a significant upregulation of *Fst* in 10- and 12-day-old KO mice, suggesting a direct relation between Cx43 and *Rb1* expression and that the cell cycle progression of SCs might be traced back to this regulator mechanism. This again supports the assumption of a sustained ‘immature’ state of SC differentiation/maturation which might cause the spermatogenic arrest at the spermatogonial level or SC-only tubules in SCCx43KO mice.

Interestingly, alterations of both Cx43 and RB1 seem to be associated with cancer development [132,133,134,135]. However, it remains the question as to how precisely Cx43 affects *Rb1*. This appears to be an interesting approach for further research, and also with regard to the pathogenesis of TGCTs as a deregulation of RB pathway has already been shown in humans [136,137,138].

Apart from the impact on SCs, SC-specific Cx43 deletion leads to great changes in GC-specific genes and to an arrested spermatogenesis at the level of spermatogonia in prepubertal mice. As expected, based on the histological findings in 10-day-old KO mice, most differences in gene expression were observed compared to their WT littermates. Predominantly, genes associated with meiosis (e.g., *Sycp1* and *Spo11*) and spermatid markers such as *Piwil1* were found to be significantly downregulated in the KO mice. In order to ensure that these alterations in gene expression were not just caused by the absence of corresponding GC populations, NGS data were normalized by means of the determined cell numbers. Even after this normalization, genes related to meiosis or haploid GC differentiation were significantly altered in KO animals. Interestingly, some genes that are specific for undifferentiated spermatogonia (e.g., *Lin28* and *Sall4*, see review [85]) also showed downregulated transcript levels in the prepubertal SCCx43KO mice, suggesting alterations already at this early GC stage.

Rode and colleagues [93] reported that during puberty, the GC component of SCCx43KO mice comprised for the most part undifferentiated spermatogonia and that in adult KO animals, spermatogenesis is arrested at the level of undifferentiated spermatogonia, observed alterations in gene expression may indicate that loss of Cx43 in SCs already influences a subpopulation of undifferentiated spermatogonia. However, apart from the different GC numbers, no obvious differences could be detected for LIN28A and SALL4 expression on protein level between the KO mice and their WT littermates. Additionally, testicular samples of adult KO and WT mice were immunostained for LIN28A and SALL4 (Figure 10). According to the previous study [93], remaining GCs in seminiferous tubules of adult SCCx43KO mice were identified as undifferentiated spermatogonia. Thus, prepubertal SCCx43KO mice seem to have significantly reduced transcript levels of *Lin28* and *Sall4* but still a sufficient amount of these for translation into detectable protein volumes.

Notably, further genes implicated in spermatogonial stem cell self-renewal and maintenance were found to be significantly altered in the SCCx43KO mice in comparison with their WT littermates. These include, for example, *Zbtb16* (also known as *Plzf*), *Taf4b* and *Atm*, which were found to be significantly downregulated, as well as *Gdnf*, which was significantly upregulated in 8-, 10- and 12-day-old SCCx43KO mice. Therefore, another hypothesis might be that Cx43 deletion causes alterations of intrinsic and extrinsic factors involved in the regulation of spermatogonial stem cell self-renewal and maintenance, resulting in the observed testicular phenotype of SCCx43KO mice.

Regarding this hypothesis, over-expression of the SC-derived factor GDNF has been reported to inhibit germ cell differentiation, resulting in an accumulation of undifferentiated spermatogonia [139], hence supporting our hypothesis, as SCCx43KO mice exhibit a spermatogenic arrest at the level of undifferentiated spermatogonia [93].

Moreover, in the hematopoietic stem cell (HSC) niche, Cx43 has already been identified as a main regulator of cell–cell communication between HSCs and stromal cells as well as for the connection with the extracellular medium via hemichannels thereby regulating HSC self-renewal [140,141,142]. In this context, it is worth mentioning that embryonic mice with a general Cx43 KO show reduced fetal liver HSCs and progenitor cells (reviewed in [141]). Additionally, the Cx43 lack in stromal cells leads to alterations in HSCs and progenitor cells in the fetal liver, preventing successful hematopoiesis (reviewed in [141]). In addition, Cx43 seems to play key roles in the neural stem cell and the skin stem cell niche (reviewed in [141]. Thus, it appears likely that Cx43 also plays a relevant role in the spermatogonial stem cell niche. Future studies are required to address this interesting issue.

Furthermore, with the aid of the created candidate gene list, promising candidate genes such as *Sohlh1* and *Sohlh2* were highlighted. NGS data of selected genes of interest could be verified using qRT-PCR and/or IHC (Figure 8 and Figure 11). The GC-specific transcription factors *Sohlh1* and *Sohlh2* play a pivotal role in spermatogonial differentiation [50,143]. As SCCx43KO mice, homozygous *Sohlh1* and *Sohlh2* KO mice are infertile and show similar phenotypes, in particular, an arrested spermatogenesis at the level of spermatogonia [50,73,74]. Thus, disturbed *Sohlh1* and *Sohlh2* gene expression appears to be an additional explanation for the testicular phenotype of the SCCx43KO mice.

In a recent study, however, fewer numbers of spermatocytes were also detected in the seminiferous tubules of *Sohlh2* KO mice, and SOHLH2 was found to be crucial for synaptonemal complex formation by regulating *Sycp1* expression during spermatogonial differentiation and therefore important for progression of meiosis [76].

Moreover, it has been shown that SOHLH proteins act as heterodimers and homodimers and directly regulate the transcription of *Gfra1*, *Sox3, Sohlh1, Sohlh2* and *Kit*, genes which are pivotal for spermatogonial development and differentiation [143]. In addition, SOHLH1 and SOHLH2 suppress genes implicated in spermatogonial stem cell maintenance/self-renewal, such as *Ret*, *Nanos2* and *Pou5f1*, affect genes implicated in RNA metabolism, for example *Lin28*, and induce the expression of genes associated with meiosis, such as *Dmrtc2* (also known as *Dmrt7*) and *Piwil1* [143]. A further notable target gene is *Stra8*, which is directly downregulated by both SOHLH1 and SOHLH2 [144]. Consequently, SOHLH proteins are believed to coordinate spermatogonial differentiation and RA-induced meiosis in cooperation with other transcription factors [144]. In this context, *Dmrt1* and *Dmrtb1* (also known as *Dmrt6*) should be mentioned because they have been assumed to play an essential role in regulating meiosis and *Stra8* expression [33,145,146,147]. Additionally, *Fignl1* has been proposed to play an important role in controlling meiosis in mice [148].

Remarkably, in the SCCx43KO mice, *Sohlh2* and *Dmrtb1* were among the most significantly altered genes and above-mentioned target genes of the SOHLH proteins (except for *Ret*, *Nanos2* and *Pou5f1)* as well as *Fignl1* were also significantly differentially expressed in the prepubertal SCCx43KO mice. Hence, a lack of Cx43 in SCs seems to have great impact on genes, which are essential for the necessary mitosis–meiosis switch.

As murine *Dmrtb1* was already investigated in 8-day-old SCCx43KO mice [9], and a successful translation of previous findings [9,33] by means of examining human testicular biopsy specimens of corresponding human pathologies [34] was possible, the current study focused on investigating *Sohlh1* and *Sohlh2*. NGS data were confirmed by qRT-PCR. In addition, SOHLH1 protein was immunolocalized in spermatogonia of WT animals, but was rarely detectable in SCCx43KO littermates, which is again consistent with NGS data. Therefore, alterations of genes required for spermatogonial differentiation, especially of *Sohlh1* and *Sohlh2* expression, might provoke the spermatogenic arrest in the SCCx43KO mice.

Furthermore, SOHLH1 has been shown to be exclusively expressed in pre-meiotic cells in the testis of rhesus monkeys [149]. Its translocation from the cytoplasm into the nucleus at the onset of puberty has been associated with the initiation of spermatogenesis [149]. This also implies a role of SOHLH1 for regulating spermatogonial differentiation in primates [149].

Taken together, *SOHLH1* and *SOHLH2* appear to be interesting candidate genes for investigating corresponding human deficiencies. At the genetic level, mutations in *SOHLH1* gene have already been associated with non-obstructive azoospermia [150,151] as well as genetic variants in *SOHLH1* and *SOHLH2* with non-obstructive azoospermia risk in Chinese men [152]. In females, too, crucial roles of *SOHLH2* have been discovered, for example in the etiology of human premature ovarian failure and in the context of ovarian cancer [153,154,155]. Nevertheless, to the best of our knowledge, no reliable data about the protein localization in human testis have been published so far. Thus, further research in this field is necessary and appears to be promising.

With regard to Cx43, the data of this study indicate that Cx43 in SCs might be involved in regulating the above-mentioned genes in GCs at a subordinate level. Concerning this matter, intercellular communication through TNTs seems to be interesting. TNTs allow the exchange of various materials, such as miRNA, proteins, vesicles derived from the endoplasmic reticulum and Golgi complex, endocytic vesicles and lysosomes, between connected cells [89,156,157]. Their biological relevance has been shown in diverse processes, including embryogenesis, stem cell differentiation and cancer progression, and Cx43 has been associated with TNTs [156,158,159,160,161,162,163,164].

As in SCs of SCCx43KO mice Cx43 is absent, it might be possible that Cx43-deficient SCs are unable to communicate with other cells through TNTs. In addition, alpha-tubulin has been identified as microtubule component of TNTs in human prostate cancer cells and *Ezr* as a constituent protein of these intercellular bridges in the rat testis [165,166]. Previous studies have shown that Cx43 directly interacts with alpha- and beta-tubulin [167,168]. For example, *Tuba3a*, *Tuba3b*, *Tuba4a* and *Tubb4b* have been found to be significantly downregulated in 8-, 10- and 12-day-old and *Tuba1c*, *Tubb1*, *Tubal3*, *Tubb4a*, *Tuba8* as well as *Ezr* in 10-day-old SCCx43KO mice, which might also suggest that cell-to-cell communication via TNTs might be disturbed in the SCCx43KO mice.

For various reasons (reviewed in [89]), investigating TNTs might be challenging but an auspicious approach.

Nevertheless, the first interesting findings about TNTs implicated in spermatogenesis have been reported in the *Drosophila* testis as well as in connection with the ectoplasmic specializations (ES) within the seminiferous epithelium of rat testes [169,170].

Additionally, Cx43, Cx45 and Cx32 have been shown to be involved in a new mechanism for rapid and efficient cell-to-cell communication by mediating the interaction of extracellular vesicles with target cells and/or the transfer of miRNAs and small molecules [89]. Apart from *Gja1* (encoding Cx43), significant alterations in *Gjc1* (coding for Cx45) and *Gjb1* (encoding Cx32) gene expression have also been detected in the SCCx43KO mice (Table S21), which might suggest that this form of cell-to-cell communication could be disturbed and might contribute to the development of the observed SCCx43KO phenotype. In order to address this question, future studies are necessary.

Moreover, the ‘classical’ functions of Cx43 as gap junction protein should not be forgotten. It seems that the reported selective and unidirectional transfer between SCs and spermatogonia [171,172,173] is altered in the SCCx43KO mice and might explain the arrested spermatogenesis in the mutant mice. Based on these permeability studies, gap junctions between adjoining SCs appear to differ in Cx composition from those between SCs and GCs [171,172,173,174]. This suggestion is supported by the impaired spermatogenesis found in SCCx43KO mice, whereas GC-specific Cx43 KO results in functional spermatogenesis and fertile mice [25,26,175]. Therefore, it has been assumed that spermatogonia form a heterotypic connexon [174,175].

Furthermore, the role of Cx43 protein itself seems to be promising. A loss-of-function study indicates that another property of Cx43, other than its ability to form gap junctions, appears to be responsible for its indispensability in germ line development [174,176]. Moreover, recent studies have reported additional functions of Cxs, such as regulating gene transcription and interacting with cell growth and cell death modulators, as well as having chemical roles [177,178,179]. Thus, further research on the different functions of Cx43 is required.

All things considered, this study provided different approaches regarding the underlying mechanisms resulting in the testicular phenotype of the SCCx43KO mice. These hypotheses need to be further investigated in future studies.

## 5. Conclusions

In conclusion, this study demonstrates that loss of Cx43 in SCs leads to significant genetic as well as morphologic changes already in prepubertal SCCx43KO mice in comparison with their WT littermates. More precisely, Cx43 in SCs seems to be essential for the normal progression of the first wave of spermatogenesis, especially for the mitosis–meiosis switch. Additionally, its expression appears to be required for successful regulation of prepubertal SC maturation. These findings seem to be in accordance with findings in corresponding human pathologies, thus the transgenic SCCx43KO mouse line proves to be a useful translational model for investigating and understanding the possible causes leading to impaired spermatogenesis and human male factor infertility.

## Figures and Tables

**Table 1:** Antibodies used for immunohistochemistry.

Protein	Primary Antibody	Host, Mono-/Polyclonal	Dilution	Secondary Antibody
**AMH**	Anti-AMH antibody [5/6] (Abcam, ab24542)	Mouse, monoclonal	1:50	Labelled Polymer-HRP Anti-mouse, ready to use
**β-galactosidase**	Anti-beta Galactosidase antibody (Abcam, ab616)	*E. coli*, polyclonal	1:1000	Labelled Polymer-HRP Anti-rabbit, ready to use
**Cx43**	Connexin 43 antibody (Cell signaling, #3512)	Rabbit, polyclonal	1:250	Biotinylated Goat Anti-Rabbit, 1:200
**LIN28A**	LIN28A (A177) antibody (Cell signaling, #3978S)	Rabbit, polyclonal	1:70	Labelled Polymer-HRP Anti-rabbit, ready to use
**SALL4**	Anti-Sall4 antibody (Abcam, ab57577)	Mouse, monoclonal	1:200	Labelled Polymer-HRP Anti-mouse, ready to use
**SOHLH1**	Kindly provided by Dr. Aleksandar Rajkovic (University of California, San Francisco)	Rabbit, polyclonal	1:500	Biotinylated Goat Anti-Rabbit, 1:200
**SOX9**	Anti-Sox9 (EMD Millipore, #AB5535)	Rabbit, polyclonal	1:1500	Labelled Polymer-HRP Anti-rabbit, ready to use

**Table 2:** Primers used for qRT-PCR.

Gene Name	Primer	Primer Sequence (5′->3′)	Accession Number/Reference	Amplicon Length (bp)
*Amh*	Forward	CCA ACG ACT CCC GCA GCT C	[9]	93
	Reverse	CTT CCC GCC CAT GCC ACT C		
*Fshr*	Forward	CTC TGG GCC AGT CGT TTT AG	NM_013523.3	150
	Reverse	GCC TCA ATG AGC ATG ACA AA		
*Gja1*	Forward	ACA GCG GTT GAG TCA GCT TG	[9]	106
	Reverse	GAG AGA TGG GGA AGG ACT TGT		
*Sohlh1*	Forward	ATG TGG CAG GGT GAT GTT CT	NM_001001714.1	146
	Reverse	GCC TGG CTC TGG TCT GTA TC		
*Sohlh2*	Forward	GCC GCT GAC CTT GGA AAA AG	NM_028937.3	121
	Reverse	GCG GGA CGT CTG AAA GTA CA		
*β-Actin*	Forward	CAC TGT CGA GTC GCG TCC	[38]	102
	Reverse	CGC AGC GAT ATC GTC ATC CA		

**Table 3:** Results from 2-way analysis of variance for testing the influence of group, time and their interaction on either germ or Sertoli cell counts.

Cells	Factor	*p*-Value
Germ cells	Group	< 0.01
	Time	< 0.01
	(Group x Time) Interaction	< 0.01
Sertoli cells	Group	< 0.01
	Time	< 0.01
	(Group x Time) Interaction	0.11

**Table 4:** Number of differentially expressed genes for each comparison with respect to the type of normalization.

Samples	Comparison	Cell Count Specific Normalization		
		None	Germ Cells	Sertoli Cells
		Number of Genes with *p* (FDR) < 0.05	Number of Genes with *p* (FDR) < 0.05	Number of Genes with *p* (FDR) < 0.05
All	KO vs. WT	11,635	33,614	10,994
	day10 vs. day8	2565	30,170	29,691
	day12 vs. day8	24,558	35,062	34,937
WT	day10 vs. day8	20,115	32,042	31,723
	day12 vs. day8	23,334	34,026	33,741
KO	day10 vs. day8	16039	532	532
	day12 vs. day8	21882	33,614	33,614
Day8	KO vs. WT	20,429	4253	4253
Day10	KO vs. WT	24,793	34,325	34,183
Day12	KO vs. WT	7126	9517	7783

KO: knockout, WT: wild type, FDR: false discovery rate.

**Table 5:** Significant gene ontology terms at all age groups.

Gene Ontology (GO) Term	Genes
DNA methylation involved in gamete generation [GO:0043046]	*Tdrd1, Tdrd9, Tdrkh, Tdrd12, Kdm1b, Fkbp6, Mov10l1, Mael, Piwil2, Pld6, Piwil4, Ddx4, Ctcfl, Prmt7, Dnmt3a, Dnmt3c, Dnmt3l, Asz1, Morc1, Tdrd5*
male meiotic nuclear division [GO:0007140]	*Sycp2, Rspo1, Suv39h2, Spo11, Spdya, Rec8, Tex14, Tdrd9, Tdrkh, Tdrd12, Tex11, Siah1a, Sgo2, Tex15, Kif18al Ing2, Hspa2, Slc2a8, Mov10l1, Mael, Mlh1, Meiob, Rad51c, Trip13, Meioc, Mybl1, Mei1, Fanca, Fignl1, Tex19.2, Ubb, Tex19.1, Ubr2, Ddx4, Catsperz, Cyp26b1, Chtf18, Brca2, Btbd18, Brdt, Ago4, Dmc1, Dmrtc2, Dnmt3c, Dnmt3l, Dpep3, Atm, Asz1*
meiotic DNA repair synthesis [GO:0000711]	*Sycp3, Sycp1, Spata22, Tex12*
synaptonemal complex [GO:0000795]	*Sycp3, Sycp1, Sycp2, Stag3, Rec8, Syn1, Smc1b, Incenp, Fkbp6, Hspa2, Hormad1, Mlh1, Rad51, Msh4, Plk1, Wapl, Polb, Lig3, P3h4, Plk1, Hormad2, Mlh3, Syce2, Mlh3, Syce1, Syce1l, Tex12, Rnf212b, Msh5*
lateral element [GO:0000800]	*Sycp3, Sycp1, Sycp2, Stag3, Rec8, Rpa1, Smc1b, Smc3, Ccdc155, Incenp, Rad51, Rad21l1, Blm, Brca2, Brca1, Mei4, Sycp2l, Xlr3b, Xlr3c, Xlr, Xlr3a*
transverse filament [GO:0000802]	*Sycp3, Sycp1, Stag3*
female meiosis sister chromatid cohesion [GO:0007066]	*Sycp3, Stag3, Rad51c*
central element [GO:0000801]	*Syce3, Sycp1, Tex11, Incenp, Six6os1, Syce2, Syce1, Tex12*
positive regulation of cell proliferation in bone marrow [GO:0071864]	*Shc1, Il6, Fgfr3, Hmga2, Mef2c, Map3k3, Lef1, Flt3lg*
nuclear meiotic cohesin complex [GO:0034991]	*Stag3, Rec8, Smc1b, Smc3, Rad21l1*

**Table 6:** List of differentially expressed genes associated with spermatogenesis.

Gene	*p*(FDR) d8	*p*(FDR) d10	*p*(FDR) d12	Localization (Cell Type)	Functions in Male Spermatogenesis
*Crabp1*	3.84 × 10^−05^	8.26 × 10^−08^	1.82 × 10^−05^	Spg [53]	Promotion of cytoplasmic degeneration of retinoic acid via the cytochrome P450 family 26 (CYP26) enzymes [54]
*Dmrtb1*	9.96 × 10^−05^	2.08 × 10^−07^	3.15 × 10^−05^	Spg, Spc (preleptotene up to pachytene stage) [33,34]	Coordination of the transition between mitosis and meiosis [33,34]
*Usp26*	2.48 × 10^−05^	3.78 × 10^−07^	6.30 × 10^−06^	Spg, decreases in Spc at leptotene/zygotene stage [55]; round and elongated Spd, localized at the blood-testis-barrier and near SC-GC interface [56]; and in human SCs and Leydig cells [57]	Does not play a decisive role in murine gametogenesis [58] Possible role in GC movement along the seminiferous epithelium [56]
*Grhl1*	3.4 × 10^−04^	5.4 × 10^−07^	2.90 × 10^−05^	Testis [59,60]	Regulating expression of genes implicated in cellular proliferation, differentiation, adhesion, and polarity [61]
*Stra8*	4.6 × 10^−04^	6.03 × 10^−07^	5.06 × 10^−06^	Spg type A and B, preleptotene and early leptotene Spc [62]	Pivotal for transition into meiotic prophase [63]
*Tex15*	1.7 × 10^−04^	6.03 × 10^−07^	1.56 × 10^−06^	Spg, early Spc, round Spd (postmeiotic reactivation) [55]	Crucial for meiotic recombination [64]
*Mei1*	3.6 × 10^−04^	6.45 × 10^−07^	1.56 × 10^−06^	Gonads; gene KO leads to arrested Spc at zygotene/pachytene stage [65,66]	Possible role for the initiation of meiotic recombination [67]
*Ovol1*	2.0 × 10^−04^	6.47 × 10^−07^	4.00 × 10^−05^	Spc, round Spd [68,69]	Regulation of meiotic pachytene progression of GCs [69]
*Sycp1*	1.4 × 10^−04^	6.47 × 10^−07^	6.56 × 10^−06^	Spc (meiosis-marker) [70]	Represents the main structural element of transverse filaments of the synaptonemal complex (a complex structure formed during meiosis) [70]
*Otx1*	4.6 × 10^−04^	8.36 × 10^−07^	1.53 × 10^−04^	Testis [59,60]	*Otx1* KO leads to a selective loss of differentiating GCs but not of spermatogonial precursors [71] *Otx1* seems to be involved in genitourinary tract development [72]
*Sohlh2*	9.52 × 10^−06^	8.36 × 10^−07^	2.87 × 10^−06^	Spg [50,73]	Required for spermatogonial differentiation [74] Promotion of spermatogonial differentiation by controlling *Kit* expression [73,75] Crucial for synaptonemal complex formation by regulating *Sycp1* expression [76]
*Taf7l*	3.81 × 10^−05^	8.36 × 10^−07^	1.50 × 10^−06^	Spg, Spc, round Spd [77]	Essential for normal sperm count and motility [77]
*Hells*	9.96 × 10^−05^	8.73 × 10^−07^	1.38 × 10^−06^	Spg, Spc (up to zygotene) [78]	Crucial for meiotic progression [78]
*Lhx8*	1.3 × 10^−04^	8.73 × 10^−07^	1.12 × 10^−04^	GCs [79]	Possibly involved in the regulation of spermatogonial differentiation [50,79] Primarily a female-specific transcriptional regulator [80]
*Mael*	4.64 × 10^−05^	8.73 × 10^−07^	1.16 × 10^−05^	Spc, round Spd [81,82]	Pivotal for spermatogenesis and transposon repression in meiosis [81]

GCs: germ cells; KO: knockout; SCs: Sertoli cells; Spg: spermatogonia; Spc: spermatocytes; Spd: spermatids. All of these genes were downregulated in the 8-, 10- and 12-day-old SCCx43KO mice.

**Table 7:** PANTHER biological process and corresponding genes.

PANTHER Biological Process	Genes
cellular component organization or biogenesis (GO:0071840)	*Patl2*
cellular process (GO:0009987)	*Spry4, Naa11, Mmp2, Birc5, Ascl2, Igf1r, Nos1, Dgkz, Mtr, Uba6, Inhbb, Zbtb42, Clgn, Parp1, Fshr, Rad51, Dpy1912, Blm, Sat2, Bcl2l2, Dab1, Kit, Usp26, Sox3, Cdc20, Rictor, Atm, Bub1b, Fignl1, Lef1, Tle3, Serpine1, Fgfr3, Wapl, Mtor, Kif18a, Xpc, Stag3, Tex19, Gdnf, Rec8, Ccne1, Ccnd1, Cib1, Smc1b, Axl, Ctcfl, Mdm2, Casp3, Foxs1, Ccnb1, Chrna7, Sohlh2, Rpl10l, Bub1, Gamt, Brca1, Sox11, Trip13, Kif17, Serpina5, Tert, Plk1, Plk4, Inha, Adgrg1, Insr, Lig4, Tssk6, Brca2, Adamts5, Rnf212, Kif2c, Il6ra, Rb1, Trf, Tyro3, Kif3a, Nek1, Sohlh1, Adamts1, Gabpa, Lhcgr, Asb9, Trdrd9, Psma8, Fmr1, Spag4*
biological phase (GO:0044848)	*Rad51, Rad51c, Meioc, Ccne1, Trip13, Rnf212*
localization (GO:0051179)	*Clgn, Sdc1, Kit, Wapl, Mtor, Axl, Trf, Tyro3, Nxf2, Fmr1, Spag4*
reproduction (GO:0000003)	*Mael, Sycp3, Rad51, Dpy1912, Piwil2, Rad51c, Piwil1, Meioc, Bub1b, Espl1, Wapl, Tex19, Rec8, Ccne1, Piwil4, Bub1, Trip13, Rnf212, Tcf15*
biological regulation (GO:0065007)	*Spry4, Mov10l1, Ascl2, Igf1r, Nos1, Dgkz, Mael, Inhbb, Fshr, Kdm1b, Neurog3, Boll, Bcl2l2, Kit, Rictor, Esr2, Meioc, Atm, Bub1b, Taf4b, Fignl1, Esr1, Lef1, Apbb1, Tle3, Fgfr3, Cpeb1, Wapl, Mtor, Mt2, Dusp6, Tex19, Ccne1, Lhx6, Ccnd1, Rffl, Axl, Ctffl, Taf1, Rbm38, Sall4, Dicer1, Ccnb1, Chrna7, Bub1, Prok2, Brca1, Trip13, Taf7l, Plk1, Plk4, Inha, Adgrg1, Insr, Lhx8, Ahr, Grhl1, Dmxl2, Rbmxl2, Il6ra, Rb1, Tcfl5, Trf, Nek1, Lhcgr, Dazl, Asb9, Tdrd9, Fmr1, Spag4, Lifr*
response to stimulus (GO:0050896)	*Spry4, Mmp4, Igf1r, Nos1, Fshr, Kit, Fgfr3, Mt2, Axl, Insr, Ahr, Lhcgr*
developmental process (GO:0032502)	*Igf1r, Gata4, Lhx6, Foxs1, Insr, Lhx8, Ovol1, Sox8*
rhythmic process (GO:0048511)	*Prok2*
multicellular organismal process (GO:0032501)	*Otx1, Mmp2, Ascl2, Nos1, Gata4, Tex19, Lhx6, Axl, Glis1, Chrna7, Hspb1, Prok2, Lhx8, Il6ra, Rb1, Cnn1, Tyro3, Fmr1*
biological adhesion (GO:0022610)	*Il6ra*
metabolic process (GO:0008152)	*Naa11, Mmp2, Mov10l1, Hsd17b1, Ascl2, Pld6, Nos1, Mtr, Mael, Clgn, Il4i1, Etnk2, Kdm1b, Neurog3, Rad51, Dpy19l2, Blm, Boll, Sat2, Gata4, Ocln, Usp26, Wbp2nl, Sox3, Cdc20, Esr2, Egr4, Meioc, Ttll5, Taf4b, Tbpl1, Esr1, Lef1, Apbb1, Tle3, Hs6st1, Uchl3, Cpeb1, Snai3, Cyp26b1, Adad1, Lhx6, Ctcfl, Taf1, Rbm38, Mdm2, Sall4, Foxs1, Gtpbp4, Dicer1, Patl2, Gamt, Brca1, Sox11, Tert, Taf7l, Lhx8, Ctsl, Ahr, Grhl1, Rbmxl2, Drosha, Gstt1, Tcfl5, Trf, Uchl1, Gabpa, Dazl, Asb9, Psma8, Fmr1*
cell proliferation (GO:0008283)	*Ccne1, Ccnd1, Ccnb1, Prok2, Lifr*
immune system process (GO:0002376)	*Dab1, Kit, Axl, Hspb1, Il6ra*

## Supplementary Materials

The following are available online at https://www.mdpi.com/2073-4409/9/3/676/s1, Figure S1: Representative negative controls for β-galactosidase (A), Cx43 (B), AMH (C) and SOHLH1 (D) immunohistochemical staining, Figure S2: PANTHER pathway analysis of candidate genes, Table S1: Animal samples used for RNA-Seq, Table S2: Candidate gene list, Table S3: NGS_DiffAnalysis_WholeData, Table S4: NGS_DiffAnalysis_WholeData_normalized, Table S5: NGS_DiffAnalysis_WholeData_normalized SC, Table S6: NGS_DiffAnalysis_WT, Table S7: NGS_DiffAnalysis_WT_normalized, Table S8: NGS_DiffAnalysis_WT_normalized SC, Table S9: NGS_DiffAnalysis_KO, Table S10: NGS_DiffAnalysis_KO_normalized, Table S11: NGS_DiffAnalysis_KO_normalized SC, Table S12: NGS_DiffAnalysis_timespecific, Table S13: NGS_DiffAnalysis_timespecific_normalized, Table S14: NGS_DiffAnalysis_timespecific_normalized SC, Table S15: NGS_GSEA_GO_WholeData_normalized, Table S16: NGS_GSEA_GO_day8_normalized, Table S17: NGS_GSEA_GO_day10_normalized, Table S18: NGS_GSEA_GO_day12_normalized, Table S19: PANTHER pathways of all significantly altered genes, Table S20: PANTHER pathways of the candidate genes, Table S21: Differential gene expression of connexins in SCCx43KO mice.
