# Supplementary material for: Loss of Cx43 in Murine Sertoli Cells Leads to Altered Prepubertal Sertoli Cell Maturation and Impairment of the Mitosis-Meiosis Switch
Source: Cells. 2020 Mar 10;9(3):676. doi: 10.3390/cells9030676 (PMC7140672; doi:10.3390/cells9030676)
Supplement: Supplementary file 1 [file cells-09-00676-s001.zip › Figure S1.docx]

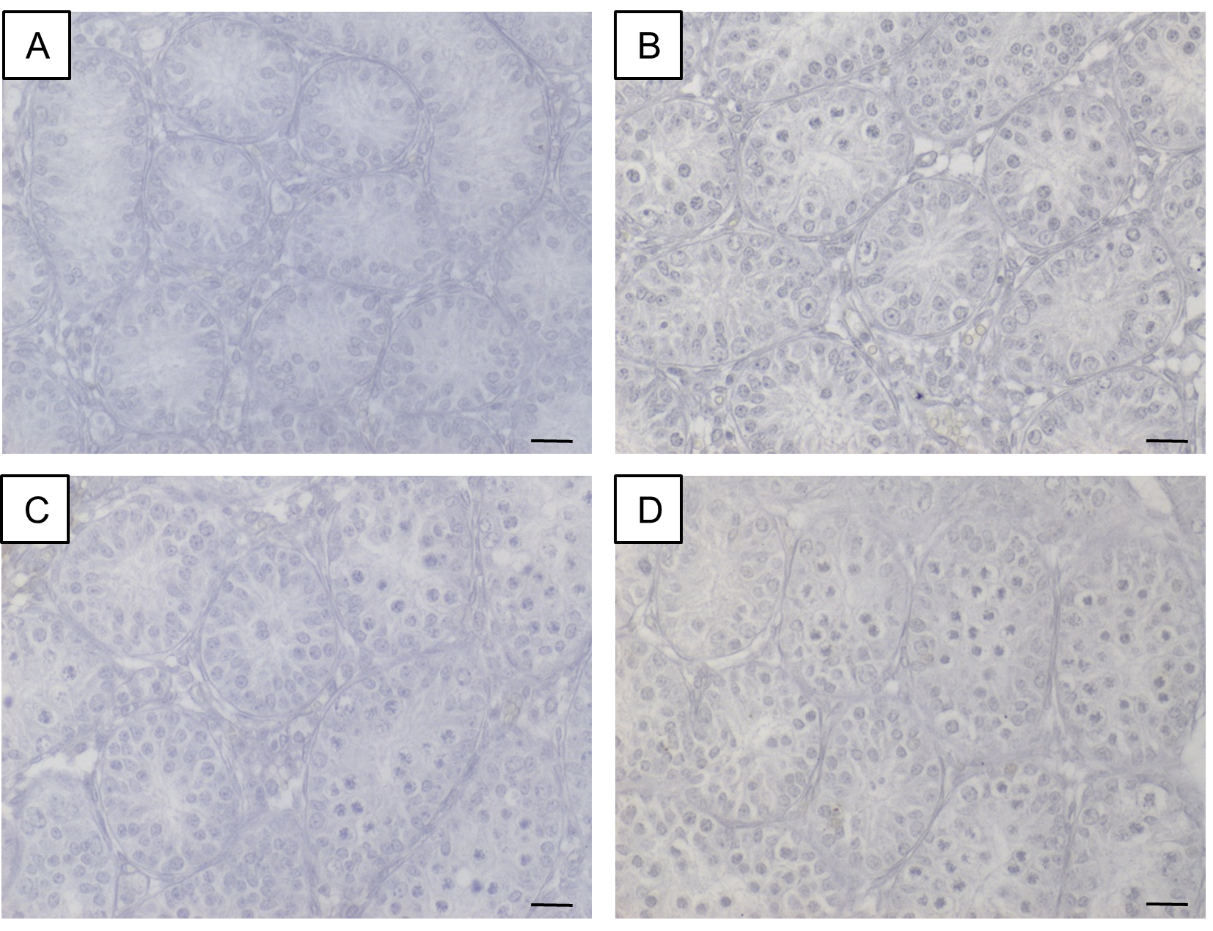


**Figure S1:** Representative negative controls for β-galactosidase (**A**), Cx43 (**B**), AMH (**C**) and SOHLH1 (**D**) immunohistochemical stainings. Shown are samples of a 12-day-old KO mouse (**A**) and 12-day-old WT mice (**B**-**C**). None of the negative controls showed any immunoreaction. AMH: anti-Müllerian-hormone, Cx43: connexin 43, KO: knockout, WT: wild type, scale bars: 20 µm, numerical aperture: 0.5.
