# Supplementary material for: Loss of Cx43 in Murine Sertoli Cells Leads to Altered Prepubertal Sertoli Cell Maturation and Impairment of the Mitosis-Meiosis Switch
Source: Cells. 2020 Mar 10;9(3):676. doi: 10.3390/cells9030676 (PMC7140672; doi:10.3390/cells9030676)
Supplement: Supplementary file 1 [file cells-09-00676-s001.zip › Figure S2.docx]

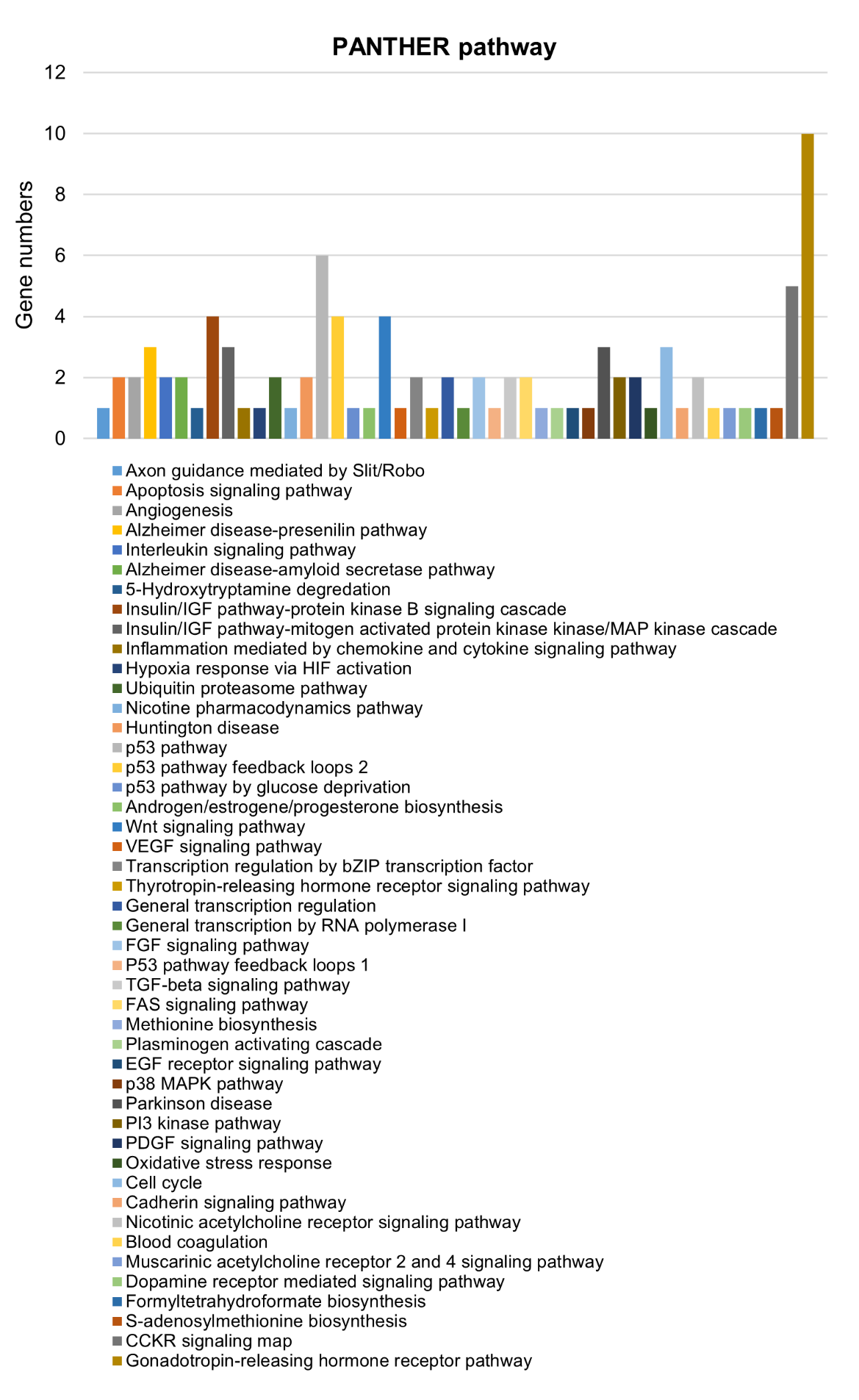


**Figure S2:** PANTHER pathway analysis of candidate genes. Gene numbers are shown in relation to corresponding PANTHER pathways.
