# Supplementary material for: Loss of Cx43 in Murine Sertoli Cells Leads to Altered Prepubertal Sertoli Cell Maturation and Impairment of the Mitosis-Meiosis Switch
Source: Cells. 2020 Mar 10;9(3):676. doi: 10.3390/cells9030676 (PMC7140672; doi:10.3390/cells9030676)
Supplement: Supplementary file 1 [file cells-09-00676-s001.zip › Table S19_PANTHER pathways of all significantly altered genes.docx]

**Table S1:** PANTHER pathways of all significant (q(FDR) <0.05) genes.

|  | **PANTHER pathways** | **Gene hits** |
| --- | --- | --- |
| 1 | Toll pathway-drosophila (P06217) | 1 |
| 2 | Axon guidance mediated by netrin (P00009) | 6 |
| 3 | Axon guidance mediated by Slit/Robo (P00008) | 3 |
| 4 | Axon guidance mediated by semaphorins (P00007) | 6 |
| 5 | Apoptosis signaling pathway (P00006) | 21 |
| 6 | Gonadotropin-releasing hormone receptor pathway (P06664) | 50 |
| 7 | Angiogenesis (P00005) | 29 |
| 8 | Alzheimer disease-presenilin pathway (P00004) | 23 |
| 9 | Alzheimer disease-amyloid secretase pathway (P00003) | 18 |
| 10 | N-acetylglucosamine metabolism (P02756) | 1 |
| 11 | Alpha adrenergic receptor signaling pathway (P00002) | 5 |
| 12 | Methylmalonyl pathway (P02755) | 4 |
| 13 | Adrenaline and noradrenaline biosynthesis (P00001) | 2 |
| 14 | Methionine biosynthesis (P02753) | 1 |
| 15 | Mannose metabolism (P02752) | 2 |
| 16 | Lysine biosynthesis (P02751) | 1 |
| 17 | CCKR signaling map (P06959) | 32 |
| 18 | Ubiquitin proteasome pathway (P00060) | 12 |
| 19 | Leucine biosynthesis (P02749) | 1 |
| 20 | p53 pathway (P00059) | 17 |
| 21 | Isoleucine biosynthesis (P02748) | 2 |
| 22 | mRNA splicing (P00058) | 3 |
| 23 | Wnt signaling pathway (P00057) | 47 |
| 24 | Heme biosynthesis (P02746) | 3 |
| 25 | VEGF signaling pathway (P00056) | 11 |
| 26 | Transcription regulation by bZIP transcription factor (P00055) | 14 |
| 27 | Fructose galactose metabolism (P02744) | 2 |
| 28 | Toll receptor signaling pathway (P00054) | 5 |
| 29 | Formyltetrahydroformate biosynthesis (P02743) | 2 |
| 30 | Tetrahydrofolate biosynthesis (P02742) | 1 |
| 31 | T cell activation (P00053) | 10 |
| 32 | TGF-beta signaling pathway (P00052) | 13 |
| 33 | TCA cycle (P00051) | 3 |
| 34 | De novo pyrimidine ribonucleotides biosythesis (P02740) | 4 |
| 35 | Plasminogen activating cascade (P00050) | 1 |
| 36 | De novo pyrimidine deoxyribonucleotide biosynthesis (P02739) | 3 |
| 37 | Parkinson disease (P00049) | 15 |
| 38 | De novo purine biosynthesis (P02738) | 9 |
| 39 | PI3 kinase pathway (P00048) | 10 |
| 40 | PDGF signaling pathway (P00047) | 21 |
| 41 | Coenzyme A biosynthesis (P02736) | 2 |
| 42 | Oxidative stress response (P00046) | 16 |
| 43 | Notch signaling pathway (P00045) | 10 |
| 44 | Nicotinic acetylcholine receptor signaling pathway (P00044) | 21 |
| 45 | Muscarinic acetylcholine receptor 2 and 4 signaling pathway (P00043) | 12 |
| 46 | Muscarinic acetylcholine receptor 1 and 3 signaling pathway (P00042) | 14 |
| 47 | Metabotropic glutamate receptor group I pathway (P00041) | 5 |
| 48 | Asparagine and aspartate biosynthesis (P02730) | 3 |
| 49 | Metabotropic glutamate receptor group II pathway (P00040) | 9 |
| 50 | Synaptic vesicle trafficking (P05734) | 5 |
| 51 | GABA-B receptor II signaling (P05731) | 8 |
| 52 | Endogenous cannabinoid signaling (P05730) | 4 |
| 53 | Ascorbate degradation (P02729) | 1 |
| 54 | Metabotropic glutamate receptor group III pathway (P00039) | 14 |
| 55 | Arginine biosynthesis (P02728) | 2 |
| 56 | JAK/STAT signaling pathway (P00038) | 3 |
| 57 | Androgen/estrogene/progesterone biosynthesis (P02727) | 4 |
| 58 | Ionotropic glutamate receptor pathway (P00037) | 11 |
| 59 | Aminobutyrate degradation (P02726) | 2 |
| 60 | Interleukin signaling pathway (P00036) | 12 |
| 61 | Interferon-gamma signaling pathway (P00035) | 6 |
| 62 | Alanine biosynthesis (P02724) | 1 |
| 63 | Integrin signalling pathway (P00034) | 25 |
| 64 | Vitamin B6 metabolism (P02787) | 1 |
| 65 | Acetate utilization (P02722) | 2 |
| 66 | Insulin/IGF pathway-protein kinase B signaling cascade (P00033) | 7 |
| 67 | ATP synthesis (P02721) | 1 |
| 68 | Insulin/IGF pathway-mitogen activated protein kinase kinase/MAP kinase cascade (P00032) | 6 |
| 69 | p53 pathway feedback loops 2 (P04398) | 11 |
| 70 | Valine biosynthesis (P02785) | 2 |
| 71 | Inflammation mediated by chemokine and cytokine signaling pathway (P00031) | 30 |
| 72 | p53 pathway by glucose deprivation (P04397) | 3 |
| 73 | Hypoxia response via HIF activation (P00030) | 5 |
| 74 | Vitamin D metabolism and pathway (P04396) | 2 |
| 75 | Vasopressin synthesis (P04395) | 4 |
| 76 | Thyrotropin-releasing hormone receptor signaling pathway (P04394) | 12 |
| 77 | Threonine biosynthesis (P02781) | 1 |
| 78 | Ras Pathway (P04393) | 16 |
| 79 | P53 pathway feedback loops 1 (P04392) | 1 |
| 80 | Oxytocin receptor mediated signaling pathway (P04391) | 11 |
| 81 | Anandamide degradation (P05728) | 1 |
| 82 | 2-arachidonoylglycerol biosynthesis (P05726) | 1 |
| 83 | Huntington disease (P00029) | 24 |
| 84 | Heterotrimeric G-protein signaling pathway-rod outer segment phototransduction (P00028) | 7 |
| 85 | Heterotrimeric G-protein signaling pathway-Gq alpha and Go alpha mediated pathway (P00027) | 22 |
| 86 | p38 MAPK pathway (P05918) | 9 |
| 87 | Heterotrimeric G-protein signaling pathway-Gi alpha and Gs alpha mediated pathway (P00026) | 24 |
| 88 | Opioid proopiomelanocortin pathway (P05917) | 5 |
| 89 | Hedgehog signaling pathway (P00025) | 3 |
| 90 | Sulfate assimilation (P02778) | 1 |
| 91 | Opioid prodynorphin pathway (P05916) | 3 |
| 92 | Glycolysis (P00024) | 3 |
| 93 | Succinate to proprionate conversion (P02777) | 4 |
| 94 | Opioid proenkephalin pathway (P05915) | 4 |
| 95 | General transcription regulation (P00023) | 13 |
| 96 | Serine glycine biosynthesis (P02776) | 1 |
| 97 | Nicotine pharmacodynamics pathway (P06587) | 7 |
| 98 | General transcription by RNA polymerase I (P00022) | 3 |
| 99 | Salvage pyrimidine ribonucleotides (P02775) | 2 |
| 100 | Enkephalin release (P05913) | 6 |
| 101 | FGF signaling pathway (P00021) | 20 |
| 102 | Dopamine receptor mediated signaling pathway (P05912) | 9 |
| 103 | FAS signaling pathway (P00020) | 8 |
| 104 | S-adenosylmethionine biosynthesis (P02773) | 1 |
| 105 | Angiotensin II-stimulated signaling through G proteins and beta-arrestin (P05911) | 5 |
| 106 | Histamine H2 receptor mediated signaling pathway (P04386) | 4 |
| 107 | Pyruvate metabolism (P02772) | 3 |
| 108 | Histamine H1 receptor mediated signaling pathway (P04385) | 12 |
| 109 | Pyrimidine Metabolism (P02771) | 3 |
| 110 | Gamma-aminobutyric acid synthesis (P04384) | 2 |
| 111 | Cortocotropin releasing factor receptor signaling pathway (P04380) | 4 |
| 112 | Endothelin signaling pathway (P00019) | 16 |
| 113 | EGF receptor signaling pathway (P00018) | 21 |
| 114 | DNA replication (P00017) | 9 |
| 115 | Cytoskeletal regulation by Rho GTPase (P00016) | 13 |
| 116 | Purine metabolism (P02769) | 1 |
| 117 | Circadian clock system (P00015) | 1 |
| 118 | Cholesterol biosynthesis (P00014) | 2 |
| 119 | Cell cycle (P00013) | 7 |
| 120 | Phenylethylamine degradation (P02766) | 1 |
| 121 | Cadherin signaling pathway (P00012) | 21 |
| 122 | Beta3 adrenergic receptor signaling pathway (P04379) | 3 |
| 123 | Blood coagulation (P00011) | 4 |
| 124 | Beta2 adrenergic receptor signaling pathway (P04378) | 9 |
| 125 | B cell activation (P00010) | 12 |
| 126 | Beta1 adrenergic receptor signaling pathway (P04377) | 9 |
| 127 | 5HT4 type receptor mediated signaling pathway (P04376) | 5 |
| 128 | Pentose phosphate pathway (P02762) | 1 |
| 129 | 5HT3 type receptor mediated signaling pathway (P04375) | 3 |
| 130 | 5HT2 type receptor mediated signaling pathway (P04374) | 13 |
| 131 | 5HT1 type receptor mediated signaling pathway (P04373) | 6 |
| 132 | 5-Hydroxytryptamine degredation (P04372) | 10 |
| 133 | 5-Hydroxytryptamine biosynthesis (P04371) | 1 |
