# Supplementary material for: Loss of Cx43 in Murine Sertoli Cells Leads to Altered Prepubertal Sertoli Cell Maturation and Impairment of the Mitosis-Meiosis Switch
Source: Cells. 2020 Mar 10;9(3):676. doi: 10.3390/cells9030676 (PMC7140672; doi:10.3390/cells9030676)
Supplement: Supplementary file 1 [file cells-09-00676-s001.zip › Table S20_PANTHER pathways of the candidate genes.docx]

**Table S2:** PANTHER pathways of the candidate genes.

|  | **PANTHER pathways** | **Gene hits** |
| --- | --- | --- |
| 1 | Axon guidance mediated by Slit/Robo (P00008) | 1 |
| 2 | Apoptosis signaling pathway (P00006) | 2 |
| 3 | Angiogenesis (P00005) | 2 |
| 4 | Alzheimer disease-presenilin pathway (P00004) | 3 |
| 5 | Interleukin signaling pathway (P00036) | 2 |
| 6 | Alzheimer disease-amyloid secretase pathway (P00003) | 2 |
| 7 | 5-Hydroxytryptamine degredation (P04372) | 1 |
| 8 | Insulin/IGF pathway-protein kinase B signaling cascade (P00033) | 4 |
| 9 | Insulin/IGF pathway-mitogen activated protein kinase kinase/MAP kinase cascade (P00032) | 3 |
| 10 | Inflammation mediated by chemokine and cytokine signaling pathway (P00031) | 1 |
| 11 | Hypoxia response via HIF activation (P00030) | 1 |
| 12 | Ubiquitin proteasome pathway (P00060) | 2 |
| 13 | Nicotine pharmacodynamics pathway (P06587) | 1 |
| 14 | Huntington disease (P00029) | 2 |
| 15 | p53 pathway (P00059) | 6 |
| 16 | p53 pathway feedback loops 2 (P04398) | 4 |
| 17 | p53 pathway by glucose deprivation (P04397) | 1 |
| 18 | Androgen/estrogene/progesterone biosynthesis (P02727) | 1 |
| 19 | Wnt signaling pathway (P00057) | 4 |
| 20 | VEGF signaling pathway (P00056) | 1 |
| 21 | Transcription regulation by bZIP transcription factor (P00055) | 2 |
| 22 | Thyrotropin-releasing hormone receptor signaling pathway (P04394) | 1 |
| 23 | General transcription regulation (P00023) | 2 |
| 24 | General transcription by RNA polymerase I (P00022) | 1 |
| 25 | FGF signaling pathway (P00021) | 2 |
| 26 | P53 pathway feedback loops 1 (P04392) | 1 |
| 27 | TGF-beta signaling pathway (P00052) | 2 |
| 28 | FAS signaling pathway (P00020) | 2 |
| 29 | Methionine biosynthesis (P02753) | 1 |
| 30 | Plasminogen activating cascade (P00050) | 1 |
| 31 | EGF receptor signaling pathway (P00018) | 1 |
| 32 | p38 MAPK pathway (P05918) | 1 |
| 33 | Parkinson disease (P00049) | 3 |
| 34 | PI3 kinase pathway (P00048) | 2 |
| 35 | PDGF signaling pathway (P00047) | 2 |
| 36 | Oxidative stress response (P00046) | 1 |
| 37 | Cell cycle (P00013) | 3 |
| 38 | Cadherin signaling pathway (P00012) | 1 |
| 39 | Nicotinic acetylcholine receptor signaling pathway (P00044) | 2 |
| 40 | Blood coagulation (P00011) | 1 |
| 41 | Muscarinic acetylcholine receptor 2 and 4 signaling pathway (P00043) | 1 |
| 42 | Dopamine receptor mediated signaling pathway (P05912) | 1 |
| 43 | Formyltetrahydroformate biosynthesis (P02743) | 1 |
| 44 | S-adenosylmethionine biosynthesis (P02773) | 1 |
| 45 | CCKR signaling map (P06959) | 5 |
| 46 | Gonadotropin-releasing hormone receptor pathway (P06664) | 10 |
